# Model-Driven Understanding of Palmitoylation Dynamics: Regulated Acylation of the Endoplasmic Reticulum Chaperone Calnexin

**DOI:** 10.1371/journal.pcbi.1004774

**Published:** 2016-02-22

**Authors:** Tiziano Dallavilla, Laurence Abrami, Patrick A. Sandoz, Georgios Savoglidis, Vassily Hatzimanikatis, F. Gisou van der Goot

**Affiliations:** 1 Laboratory of Computational Systems Biotechnology, Ecole Polytechnique Fédérale de Lausanne (EPFL), Lausanne, Switzerland; 2 Global Health Institute, Ecole Polytechnique Fédérale de Lausanne (EPFL), Lausanne, Switzerland; 3 Swiss Institute of Bioinformatics, Lausanne, Switzerland; Princeton University, UNITED STATES

## Abstract

Cellular functions are largely regulated by reversible post-translational modifications of proteins which act as switches. Amongst these, S-palmitoylation is unique in that it confers hydrophobicity. Due to technical difficulties, the understanding of this modification has lagged behind. To investigate principles underlying dynamics and regulation of palmitoylation, we have here studied a key cellular protein, the ER chaperone calnexin, which requires dual palmitoylation for function. Apprehending the complex inter-conversion between single-, double- and non- palmitoylated species required combining experimental determination of kinetic parameters with extensive mathematical modelling. We found that calnexin, due to the presence of two cooperative sites, becomes stably acylated, which not only confers function but also a remarkable increase in stability. Unexpectedly, stochastic simulations revealed that palmitoylation does not occur soon after synthesis, but many hours later. This prediction guided us to find that phosphorylation actively delays calnexin palmitoylation in resting cells. Altogether this study reveals that cells synthesize 5 times more calnexin than needed under resting condition, most of which is degraded. This unused pool can be mobilized by preventing phosphorylation or increasing the activity of the palmitoyltransferase DHHC6.

## Author Summary

The endoplasmic reticulum (ER) is the largest intracellular organelle of mammalian cells. It is responsible for many fundamental cellular functions, such as folding, quality control of membrane and secreted protein, lipid biosynthesis, control of apoptosis and calcium storage. Recent studies have shown that many ER membrane proteins are lipid modified. We therefore hypothesized that palmitoyltransferases, the enzymes responsible for this modifications, act as a regulator of the mammalian ER, controlling the function of a network of key proteins through reversible acylation. In this work we combine computational methods with experimental determination of parameters to study the mechanisms and properties of ER palmitoylation, using as a model the palmitoylation of the ER protein calnexin. The systematic analysis of the mathematical model, built and calibrated with the help of experimental data, shows that Calnexin palmitoylation leads to a 9-fold increase in half-life and that a long delay separates synthesis from palmitoylation in unstimulated cells. Surprisingly during this delay, 75% of synthesized calnexin is degraded before being palmitoylated. We hypothesize that this unexpected apparent inefficiency is a design principle that provides the cell with a means to post-translationally tune the calnexin content.

## Introduction

Reversible post-translational modifications of proteins allow cells to regulate processes in time and in space [1–5]. Amongst these, S-palmitoylation is unique in that in confers hydrophobicity to proteins by covalent attachment of a fatty acid chain to cysteine residues [6, 7] [8]. In the cytoplasm, this enzymatic reaction is mediated by palmitoyltransferases of the DHHC family and reversed by acyl protein thioesterases (APTs) [6, 7, 9]. Recent large-scale palmitoyl-proteome profiling studies have jointly revealed that hundreds of proteins, with major cellular functions, undergo this lipid modification in mammalian cells [10–14]. Although S-palmitoylation was identified more than 30 years ago, our understanding of this modification, its dynamics, its regulation and its consequences on protein properties, is still rudimentary.

The aim of this paper is to study the palmitoylation events, and their dynamics, occurring on a key component of the endoplasmic reticulum (ER), the type I transmembrane protein calnexin. Here we report the step-by-step design and output analysis of the first model of a palmitoylation network. Besides studying palmitoylation, another valuable objective of this work has been the estimation of system parameters which cannot be estimated by simple experiments, such as the time required for calnexin to get double palmitoylated or the half life of the palmitoylated species, but instead, they require the consideration of the system as a whole.

Calnexin is best known for its function as a lectin-like chaperone involved in the folding of glycosylated proteins in the lumen of the ER [15]. It is also involved in regulating calcium homeostasis at ER-mitochondria contact sites [16]. More recently, we have found that calnexin can act as an ER sensor, modulating the transcriptional response of cells to EGF in an ER-stress dependent manner [17]. Importantly, the ability of calnexin to assist folding of newly synthesized proteins, to control calcium signalling and to modulate the EGF signalling response, all require its palmitoylation [16–19].

Calnexin is composed of a large well-folded N-terminal luminal domain that carries the chaperone activity [20]. It is followed by a single transmembrane domain that terminates with two cysteine residues at positions 502 and 503, which are the sites of palmitoylation [18, 19]. Even though the ER contains numerous DHHC enzymes [21], calnexin palmitoylation is mediated exclusively by DHHC6 [19]. The palmitoylation sites are followed by a 90 residue cytosolic tail that is predicted to be disordered and contains multiple phosphorylation sites [20]. This cytosolic domain has multiple functions. It allows association of calnexin with the ribosome translocon complex [19, 22] but can also be proteolytically released following specific stimuli such as apoptotic drugs [23] or EGF [17]. In the latter case, the released cytosolic tail binds to PIAS3 (Protein Inhibitor of Activated STAT) and thereby promotes EGF-induced STAT3-mediated transcriptional response to EGF [17].

Since calnexin has two sites of palmitoylation, it can exist in cells under different forms: non-modified, palmitoylated on one site or the other, or on both. To grasp the full complexity of the system, we combined mathematical modelling of the system with experimental determination of the kinetics of palmitate acquisition and turnover, and of protein degradation for wild type (WT) and palmitoylation-deficient mutants. We set up a general model consisting of ordinary differential equations (ODE), describing the dynamic transitions between states/species of the network. We defined the topology of our network as a combination of well-known inter-convertible cycles described by Goldbeter and Koshland [24]. Combinations of such subunits have been used to successfully describe biological systems in which proteins undergo multiple covalent modifications, especially in the context of protein kinase signalling networks [25–27]. Since palmitoylation has, as phosphorylation, the potential to control protein function in a switch-like manner, a similar approach appeared suitable to model calnexin palmitoylation.

Mathematical modelling allowed us to access an unprecedented level of understanding of the dynamics and the complexity of inter-convertible species of the same protein undergoing various post-translational modifications on multiple sites and access key parameters that are not directly measurable through experimentation. We could in particular estimate the half-life of single or dual palmitoylated calnexin, the off-rate of palmitate from a specific site upon occupancy of the other site or the time that separates calnexin synthesis from palmitoylation events, all together revealing the existence of a regulatory system that allows cells to post-translationally control the cellular levels and thus activity of this key ER chaperone.

## Results

### Mathematical model of calnexin palmitoylation

**Fig 1:**
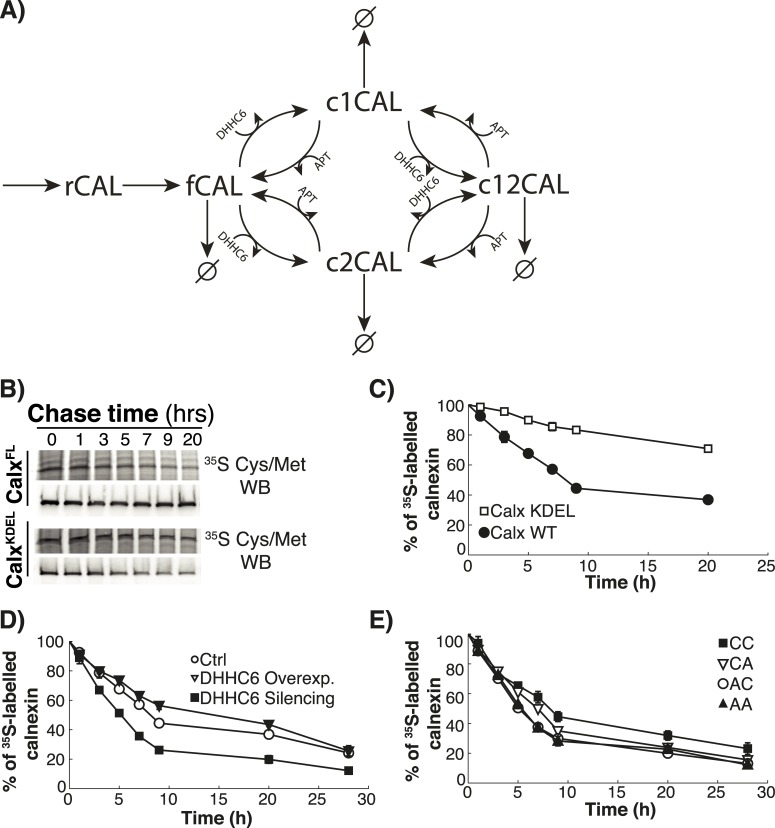
Calnexin palmitoylation model and kinetics of decay. **A:** Calnexin is first synthesized (rCAL). During the folding process the protein assumes the proper conformation (fCAL), which then can be palmitoylated twice by DHHC6. The first palmitoylation can occur on either of the two sites leading to c1CAL or c2CAL. The second palmitoylation events leads to c12CAL. We assume that both palmitoylation steps are reversible. Degradation can occur from all states. **B-E:** HeLa cells were transfected or not for 24h with calnexin-WT-HA, HA-calnexin-KDEL, calnexin-CA-HA, calnexin-AC-HA, calnexin-AA-HA and with or without DHHC6-myc, or additional transfected for 72h with DHHC6 siRNA. Cells were incubated 20 min pulse at 37°C with ^35^S-methionine/cysteine, washed and further incubated for different times at 37°C. Calnexin was immunoprecipitated and analyzed by SDS-PAGE. Autoradiography **(B)** and western blotting were quantified using the Typhoon Imager (Image QuantTool, GE healthcare). **C:** Decay profile of WT calnexin (Calx-WT) and of a calnexin mutant in which the transmembrane and cytosolic domain were removed and replaced by the KDEL sequence for ER retention (Calx-KDEL). Errors correspond to standard deviations (n = 7 for Calx-WT, n = 3 for Calx-KDEL). **D:** Decay profile of WT calnexin was observed under normal condition (Ctrl) and after overexpression (DHHC6 Overexpression) or silencing (DHHC6 Silencing) of DHHC6. Errors correspond to standard deviations (n = 7, 3, 3 for Crtl, DHHC6 overexpression and silencing respectively). **E:** Decay profile of WT calnexin (Calx-WT) and different mutants, in which site c1 (Calx-AC), site c2 (Calx-CA), or both palmitoylation sites (Calx-AA) were mutated. Errors correspond to standard deviations (n = 8, 3, 3, 3 for Calx-WT, AC, CA and AA respectively).

Calnexin was recently shown to rely on palmitoylation to perform its major functions [17–19, 28]. This raises the question as to which percentage of the total calnexin population is at a given time palmitoylated. At present, no reliable method enables to determine the percentage of a protein that is palmitoylated and to differentiate single from double palmitoylation. To estimate the species distribution and understand the dynamics of the inter-conversion between them, we therefore developed a mathematical model of the calnexin palmitoylation cycle. Modelling was performed as an open system, including protein synthesis, and degradation of all species (S1 Table, Fig 1A).

Calnexin is synthesized by ER-associated ribosomes and inserted into the ER membrane (represented by rCAL). Presumably already co-translationally, the calnexin luminal domain undergoes folding, a process that is very efficient as discussed below. The cytosolic tail of calnexin is predicted to be highly disordered (IUPRED [29], http://iupred.enzim.hu/). fCAL represents folded but non-palmitoylated calnexin. This species can be modified on the first or second palmitoylation site leading to c1CAL and c2CAL, respectively. Single palmitoylated species can acquire a second palmitate both leading to c12CAL (Fig 1A). The palmitoyltransferase DHHC6 catalyses the palmitoylation reaction for each site [19]. Depalmitoylation, which requires acyl protein thioesterases [8], the identity of which remain to be established for calnexin, can occur from both sites.

The individual palmitoylation and depalmitoylation steps were assumed to follow irreversible Michaelis-Menten kinetics, similar to the kinetics used in signalling network models [30]. These kinetics were determined using the total quasi steady state approximation (tQSSA) to a mass action model [31–33] reported by Pedersen et al. for the study of phosphorylation and dephosphorylation cycles [34, 35]. This approach included a step-by-step application of tQSSA to a variety of biochemical reactions and contained a formulation of tQSSA for systems with competitive inhibition. As Gunawardena and colleagues pointed out [36] this avoids any ad hoc assumption inherent in the common Michaelis-Menten equations and takes into account sequestration effects when enzymes have multiple substrates. In these systems, a single enzyme can catalyse the same reaction on multiple substrates, which is conceptually similar to multiple sites on the same substrate. The equations could therefore be adapted to palmitoylation and depalmitoylation of the two cytoplasmic calnexin cysteines.

Although this approximation is valid for a very wide range of enzyme-substrate concentrations [34], in the case of calnexin palmitoylation, the validity of the Michaelis-Menten equations was ensured by the relative concentration of DHHC6 with respect to calnexin. Based on quantitative proteomic studies, the ratio of DHHC6-to-calnexin is indeed in the order of 1:500 [37–39]. Since the two sites might not be equivalent and since rates might depend on the occupancy of the neighbouring site, we introduced different Km values for each palmitoylation and depalmitoylation step. Also since the same enzyme catalyses the palmitoylation of both sites, a competition term between the two sites was implemented in the enzymatic kinetics as described in [35]. A similar competition term was introduced for depalmitoylation. The model also includes degradation rates for each species, with different first-order rate constants. The description of the rate expressions, the definition of the parameters, and the assumptions used in the development of the model are described in detail in the Expanded View (S1 Fig and S1–S3 Tables).

### Experimental analysis of the effect of palmitoylation on calnexin stability

Sets of experimental data were generated to calibrate and test the predictability of the model. Since degradation of all species was included in the model, we first monitored protein degradation kinetics using ^35^S Cys/Met metabolic pulse-chase experiments. This requires immuno-precipitation of calnexin which we performed either using a polyclonal antibody against the C-terminus to follow the endogenous protein, or using an anti-HA antibody to follow transiently transfected WT and calnexin-HA mutants. WT calnexin, endogenous or HA-tagged, showed identical biphasic decays, with a t_1/2_ of ca. 8h (shown only for calnexin-HA Fig 1B–1E).

The faster initial decay made us wonder what the contribution is of the luminal domain, the largest domain of calnexin, in shaping this decay curve. We generated a truncated version of calnexin consisting of the luminal domain fused to the KDEL sequence to ensure ER localization. HA-calnexin-KDEL was far more stable than the full-length protein (Fig 1B and 1C). In particular there was no decay at early time points indicating that the luminal domain undergoes efficient folding. Thus the initial decay phase observed for the full-length protein must be dictated by transmembrane domain and/or the cytosolic tail of calnexin.

Decay of full-length calnexin was accelerated by silencing the DHHC6 enzyme (Fig 1D), or by mutating one or both of the palmitoylation sites (Fig 1E). In reverse, calnexin decay was slowed down by overexpression of DHHC6 (Fig 1D). Based on these metabolic 20 min pulse-chase experiments, the apparent half-life of WT calnexin is ca. 8h, while that of non-palmitoylated calnexin, obtained either through enzyme silencing or site mutation, is ca. 5h.

### Experimental determination of palmitate turnover on Calnexin

**Fig 2:**
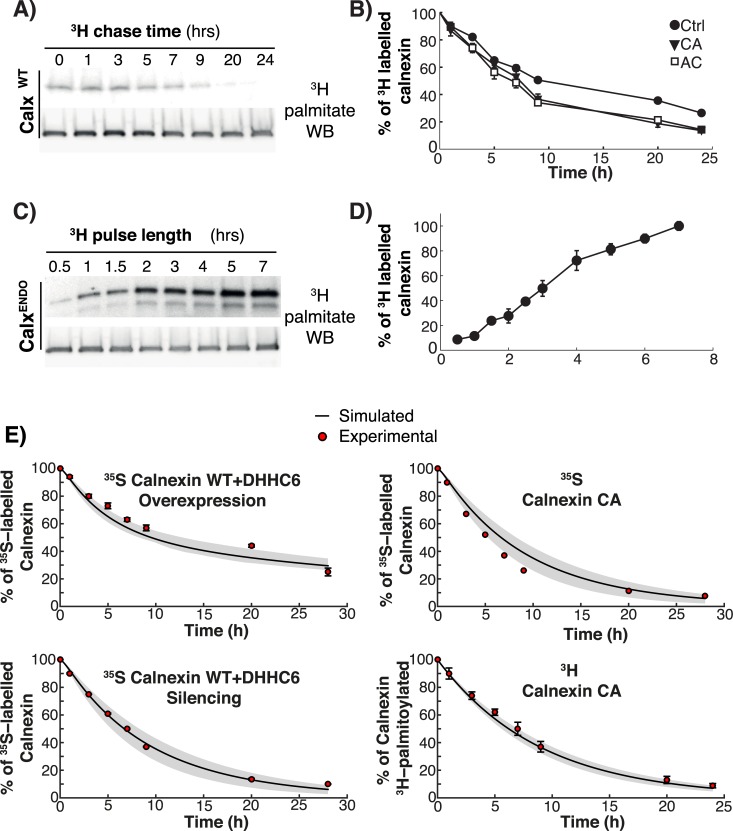
Experiments and modelling of Palmitoylation/depalmitoylation of calnexin. **AB**: HeLa cells were transfected for 24h with calnexin-WT-HA, calnexin-CA-HA, or calnexin-AC-HA. Cells were incubated with ^3^H-palmitic acid for 2h, washed and further incubated for different times at 37°C in complete medium prior to immunoprecipitation using anti-calnexin or anti-HA antibodies. Immunoprecipitates were split into two, run on SDS-PAGE and analyzed either by autoradiography (^3^H-palmitate) or Western blotting (anti-HA). Autoradiograms were quantified using the Typhoon Imager (Image QuantTool, GE healthcare). Errors correspond to standard deviations (n = 3). **CD:** HeLa cells were incubated with ^3^H-palmitic acid for different times at 37°C, washed prior to immunoprecipitation using anti-calnexin antibodies. Immunoprecipitates were split into two, run on SDS-PAGE and analyzed either by autoradiography (^3^H-palmitate) or Western blotting (anti-calnexin). Errors correspond to standard deviations (n = 4). **E:** Validation of the model output though comparison of the *in silico* experiments with experimental data that was not used in the calibration of the model. Solid line is the mean of the simulations of 382 models; the shaded area is defined by the first and the third quartile of the simulations of 382 models (details of the *in silico* labelling experiment can be found in the Supplemental Information).

To further fuel the mathematical model of the palmitoylation cycle with experimental data, we determined the turnover of the palmitate moiety once attached to calnexin. To do so, we performed pulse-chase experiments using ^3^H-palmitate, followed by immunoprecipitation of calnexin. Following a 2 h ^3^H-palmitate pulse, loss of ^3^H-palmitate from WT calnexin occurred with an apparent half-life of ca. 8 hrs and was somewhat accelerated in single cysteine mutants (Fig 2A and 2B). We also monitored the kinetics of incorporation of ^3^H-palmitate into calnexin (Fig 2C and 2D). This was performed in the presence of cycloheximide, to prevent synthesis of new proteins during the labelling time. Degradation was not prevented with any drug. Palmitate incorporation increased as a function of time and did not reach a plateau within the 7 h time frame of the experiment. Experiments were kept within this time frame to avoid toxic/indirect effects of prolonged inhibition of protein synthesis.

Altogether these experiments show that WT calnexin can undergo palmitoylation hours after it has been synthesized and that turnover of palmitate is very slow.

### Calibration and validation of the mathematical model

A subset of the above ^35^S Cys/meth and ^3^H-palmitate pulse-chase experiments were used to calibrate the system, i.e. for parameter estimation, namely: decays of WT, the AC and the double AA mutants, incorporation of ^3^H-palmitate into WT calnexin, ^3^H-palmitate turnover for WT and the CA mutant. Since there is not a unique set of parameters that fits such a dataset, we employed a stochastic optimization method, which allowed us to generate a population of models, each having different combinations of parameter values while all being consistent with the calibration experiments. From a population of 10’000 models, we selected 382 models that best fitted the experimental data used as objective function (see Expanded View, results of the fitting on the calibration dataset are shown in S2A–S2F Fig). The pool of selected models was subsequently used for the simulations and analyses. Note that all generated predictions were obtained by simulating each model independently. The outputs of all the models were then averaged and the standard deviation with respect to the mean was used as a measure of the variability among the different models (S2A–S2L Fig).

The remaining set of experiments were used to validate the output of the model, i.e. test its predictability (see Expanded View), namely the WT calnexin decay upon DHHC6 silencing or overexpression, the decay of the single CA cysteine mutant as well as palmitate turnover for this mutant. As illustrated in Figs 2E and S2G–S2L, the predictions were in close agreement with the experimental data, indicating that the model reliably describes the events of the calnexin palmitoylation/depalmitoylation cycle.

### Predicted species distribution of calnexin

**Fig 3:**
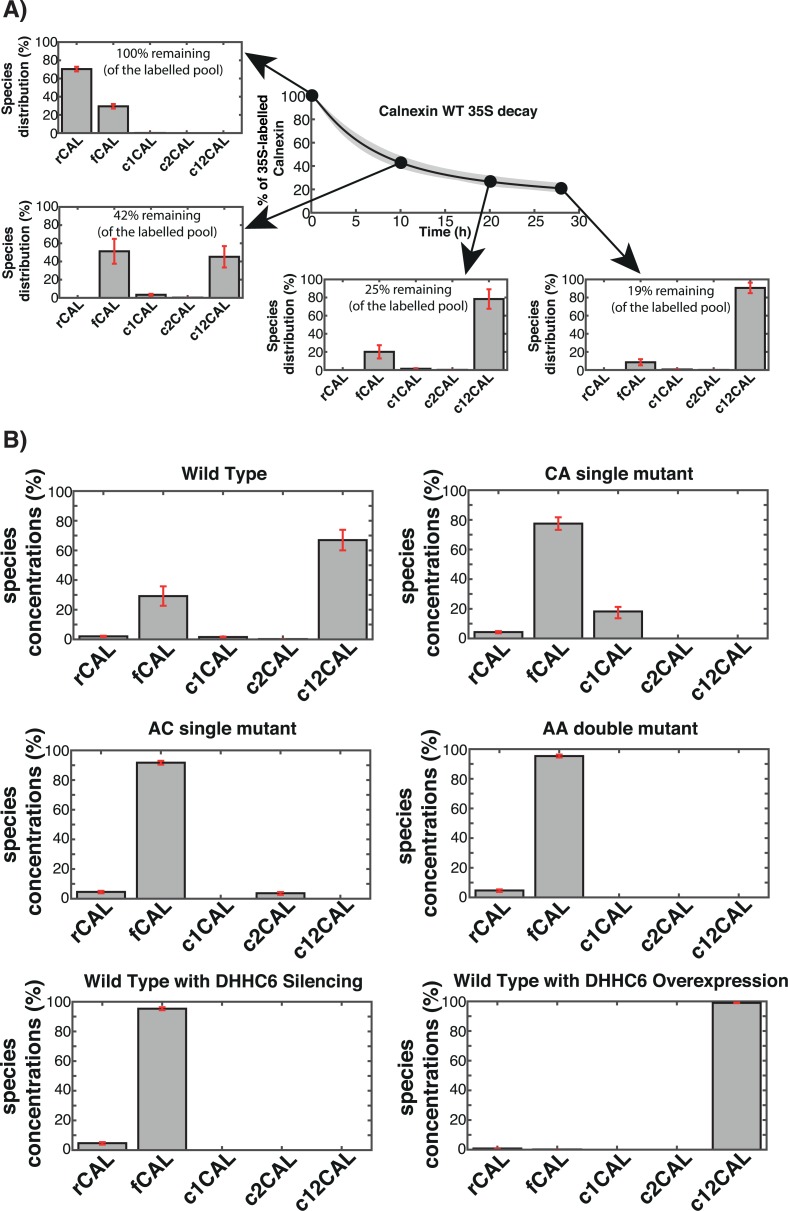
*In silico* analysis of the calnexin species distribution. **A:** We replicated *in silico* the ^35^S pulse-chase experiment on WT calnexin. During the experiment we monitored the relative distribution of calnexin in the different palmitoylation states. Solid line is the mean of the simulations of 382 models; error bars are defined by the first and the third quartile of the simulations of 382 models (details of the *in silico* labelling experiment can be found in the Expanded View). **B:** The model was used to predict the steady state distribution of WT calnexin and of the mutants in the cell. rCAL correspond to calnexin during the synthesis, fCAL represent folded calnexin while c1CAL and c2CAL denote the two single palmitoylated species. c12CAL represents the double palmitoylated calnexin. Error bars correspond to first and third quartile of the simulations of 382 models (details of the *in silico* labelling experiment can be found in the Supplementary Information).

The model was first used to determine the distribution, and the evolution thereof, of the 5 palmitoylation species during the ^35^S-Cys/Meth pulse-chase experiments. We set up an *in-silico* 20 minutes labelling experiment (see Expanded View, S3 Fig) and calculated the relative concentrations of calnexin in the different palmitoylation states at different time points of the chase (Fig 3A). At the end of the metabolic pulse (t = 0), the population is predicted to be exclusively of non-palmitoylated, ca. 25% of which are already folded. Ten hours after the pulse, the entire calnexin population was predicted to be folded, as can be expected, but, more unexpectedly, ca. 50% was still non-palmitoylated (Fig 3A). Almost the palmitoylated species, the dually-palmitoylated was predicted to be the most populated, with barely and single palmitoylated species. As chase time proceeded, the non-palmitoylated species decreased again to the benefit of the dual palmitoylated form, which approached 90% at the end of the chase period (Fig 3A). Note that distributions are expressed as percentages of the remaining population, not of the initial population, which decreased by 80% between the beginning and the end of the chase period.

This analysis indicates that the initial faster phase of degradation occurs when most of calnexin is non-palmitoylated. Once a significant percentage of the population becomes acylated, the rate of decay drastically decreases, confirming a stabilizing effect of palmitoylation.

We next determined the distribution of WT calnexin at steady state, since it may differ from the distribution at the end of our chase period. At steady state in our Hela cells and under our experimental conditions, the model predicts that ca. 70% of calnexin is dual-palmitoylated and the remaining population is free of palmitate. The prediction that the great majority of cellular calnexin is palmitoylated is consistent with our previous experimental evidence [19]. We noted that palmitoylated calnexin poorly migrates in 2D gels. We subsequently compared the calnexin signal by western blotting on 2D gels of control cells vs. cells silence for the DHHC6 palmitoyltransferase, and found that the signal was increased by ≈9 fold. This value is rather qualitative given the non-linearity of western blotting, but clearly indicated that the majority of calnexin is palmitoylated at steady state.

We next utilised the model to estimate the steady state distribution of calnexin species in cells over-expressing DHHC6 and found that the dually-modified population increased to almost 100% while it fell to zero upon silencing of the enzyme (Fig 3B), as expected. By regulating DHHC6 amounts and/or activity, cells thus have the potential to control the percentage of calnexin that is dually-palmitoylated, and thus functional.

**Fig 4:**
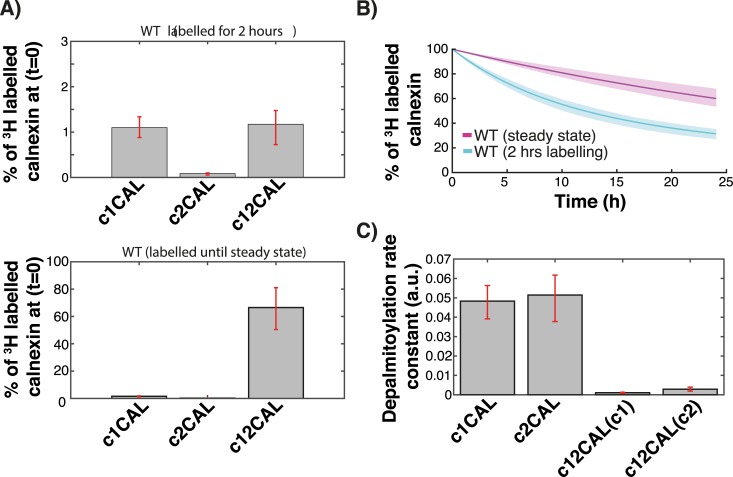
Prediction of depalmitoylation kinetics. **A:** WT calnexin was labelled with 3H-palmitate in silico either for two hrs or to reach the steady state distribution of palmitoylation species. **B:** The kinetics of palmitate loss starting from the two distributions in A were determined. The solid lines represent the means of the simulations of 382 models and the shaded areas are defined by the first and the third quartile of the simulations of 382 models (details of the *in silico* labelling experiment can be found in the Expanded View). **C:** The rate constants for depalmitoylation from c1CAL, c2CAL and c1c2CAL where determined. Error bars correspond to first and third quartile of the simulations of 382 models.

Consistent with the low population of single palmitoylated species for WT calnexin, determination of the steady state species distribution of the CA and the AC calnexin mutants revealed that >80% of the molecules are non-palmitoylated. This indicates that to obtain a significant population of palmitoylated calnexin, two sites are necessary. That the single palmitoylated species do not get significantly populated was initially unexpected given the relatively small difference in the apparent palmitate turnover rates of WT and single cysteine mutants (Fig 2B). To understand this apparent inconsistency, we used the model to predict the distribution of the species labelled during the 2 hrs ^3^H-palmitate pulse (corresponding to t = 0 in Fig 2B) (S4 and S5 Figs, Supporting information). *In silico*, ^3^H-palmitate labelling shows that in 2 hrs, only a minute percentage of the total WT population–less than 3%–undergoes labelling (S5 Fig). This is consistent with the fact that 70% of the steady state population is dual palmitoylated at steady state and can thus not be further modified upon addition of ^3^H-palmitate. Moreover, Fig 2D indicates that palmitoylation can be very slow, since some proteins undergo palmitoylation 7 h or more after having been synthesized (Fig 2D). Thus Fig 2B represents the loss of ^3^H-palmitate from a population of ^3^H-palmitate labelled species composed roughly of 50% c1CAL and 50% c12CAL (Fig 4A, top panel), jointly representing just 3% of the total population.

To estimate the rate of palmitate release from dually-palmitoylated calnexin (c12CAL), which is not readily accessible experimentally, we determined what the ^3^H-palmitate decay would be if the starting point was that of steady state palmitoylation, i.e. 70% calnexin dually labelled (Fig 4A, bottom panel). Under these conditions, the loss of palmitate was significantly slower (Fig 4B). In fact, we measured an average turnover rate for palmitate of ~32h, which is almost 3 times slower than the apparent turnover rate estimated by our 2 hrs labelling experiment. This analysis indicates that the rate constant of palmitate removal from a given site depends on the occupancy of the second site. Our predictions indeed indicate that, in situations of single site occupancy, the rate constants of loss of palmitate from site 1 are drastically higher than the rate constants of loss from either site in the situation of double occupancy (Fig 4C). Thus, single palmitoylated species do not get significantly populated as compared to the double palmitoylated one, because they lose their palmitate at a far higher rate.

Altogether this analysis indicates that two sites are required to stably palmitoylated calnexin, because double occupancy drastically slows down depalmitoylation.

### Estimated half-lives of palmitoylated calnexin species

**Fig 5:**
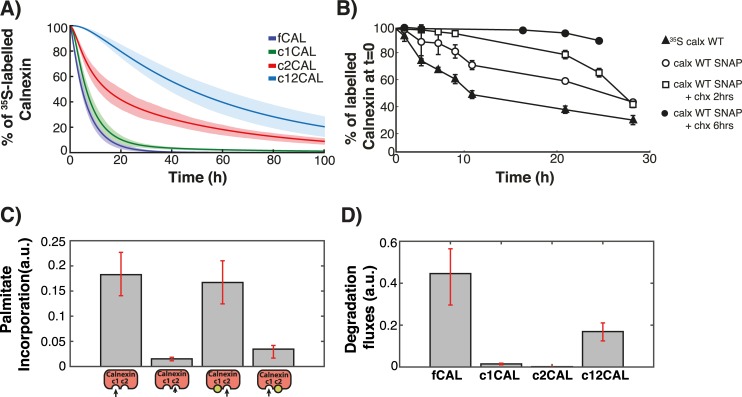
Kinetics of calnexin palmitoylation and stability. **A:** The decay curves were predicted for the different palmitoylation species: fCAL represent folded calnexin, c1CAL and c2CAL the single palmitoylated species, c12CAL double palmitoylated calnexin. Solid line is the mean of the simulations of 382 models; shaded area is defined by the first and the third quartile of the simulations of 382 models (details of the *in silico* labelling experiment can be found in the Expanded View). **B:** Decay profiles were determined for WT calnexin either by ^35^S Cys/Meth labelling (^35^S calx WT) and by SNAP labeling (Calx WT SNAP). **C:** The model was simulated until it reached steady state and the values of the palmitoylation rates under steady state conditions were plotted. Error bars correspond to first and third quartile of the simulations of 382 models (details of the *in silico* labelling experiment can be found in the Expanded View). **D:** The model was simulated until it reached steady state and the values of the degradation rates under steady state conditions were plotted. Error bars were determined as in **(C).**

^35^S Cys/Met pulse-chase experiments of WT and cysteine mutants indicate that palmitoylation has a stabilizing effect and that the half-life of palmitoylation deficient calnexin is ca. 5 hrs. They however do not allow direct identification of the half-life of single or dually palmitoylated calnexin. We therefore made use of the model to predict the decay kinetics of each of the calnexin species (Table 1 and Fig 5A). Palmitoylation of site 1 has a mildly stabilizing effect, the half-life of c1CAL being ca. 6.5 hrs (Fig 5A). Palmitoylation of site 2, whether site 1 is or not modified, leads to a spectacular stabilization, with dual-palmitoylated calnexin having a predicted half-life of 45 hrs (Table 1 and Fig 5A).

**Table 1:** Half-life of the different calnexin species. The standard deviation is estimated from the 382 models (see Supplementary material).

Species	Half-Life
**fCAL**	5.0 ± 1.5
**c1CAL**	6.5 ± 1.8
**c2CAL**	14.5 ± 1.8
**c12CAL**	46.0 ± 11.2

We sought for an experimental confirmation for this almost 10-fold increase in protein stability. At steady state, 70% of calnexin is predicted to dually-modified (Fig 3B). To assess the half-life of this population, we chose to monitor the stability of calnexin tagged with SNAP, a widely used protein that self-labels when incubated with *O*^6^-benzylguanine derivatives [40]. We labeled cells for 30 min with SNAP-cell-TMR-star, a red fluorescent substrate of SNAP, in order to label the entire, steady state, population of calnexin-SNAP. Following different periods of chase, cells were harvested and the level of fluorescent calnexin was monitored by SDS-PAGE and fluorescence scanning. As shown in Fig 5B, the decay of calnexin-SNAP-TMR-star was slower than that observed by ^35^S pulse-chase, yet still biphasic. At t = 0 of SNAP-cell-TMR-star labeling, the population of calnexin-SNAP molecules have different”ages”, the “youngest” having just been synthesized. We therefore tested whether pretreatment of cells with cycloheximide for different times, to block protein synthesis, would affect the apparent stability of the SNAP-cell-TMR-star labeled population. A 2 hrs cycloheximide pretreatment already led to an increase in apparent half-life of the population (Fig 5B). We extended the pretreatment to 6 h, so that all calnexin molecules would be at least 6 h “old”. With this treatment, no decay was observed for the first 24 hrs, and this was followed by a slow decline, leading to an apparent half-life of ca. 47 hrs (Fig 5B).

Altogether, the mathematical modeling and the calnexin-SNAP tagged decay analysis indicate that palmitoylation of the two juxtamembranous sites leads to a dramatic increase in the stability of calnexin.

### Cooperativity between the two calnexin palmitoylation sites

The analysis of the steady state distribution of WT and cysteine mutants (Fig 3B) indicates that two sites are required for stable palmitoylation and suggest that there might be cooperativity between sites. We therefore estimated the rates of palmitate incorporation at each site, depending on whether the other site was occupied or not. As shown in Fig 5C, palmitoylation on site 1 is predicted to be drastically more efficient than on site 2. However, if site 1 is already occupied, site 2 is readily modified. In marked contrast, if site 2 is occupied, site 1 by being positioned between the transmembrane and the palmitoylated site 2 might be less accessible to the enzyme. This reaction flux analysis indicates that site 1 is preferentially modified and when this has occurred, site 2 is rapidly acylated, indicating positive cooperatively between sites 1 and 2.

### Efficient degradation of calnexin is predicted to require depalmitoylation

We next simulated the degradation fluxes of the different calnexin species (Fig 5D). Even though nearly 70% of the protein is dual-palmitoylated at steady state (Fig 3B), the degradation flux was highest for the non-palmitoylated state. Doubly-palmitoylated calnexin did undergo degradation, but at a 3 to 4 times slower rate, which is due to the 3 to 4 times lower values of the degradation rate constants (by parameter estimation using the corresponding experimental information, S2 Table). Therefore, efficient degradation of calnexin appears to require prior depalmitoylation by thioesterases.

### Estimation of the lag-time separating calnexin synthesis from palmitoylation

**Fig 6:**
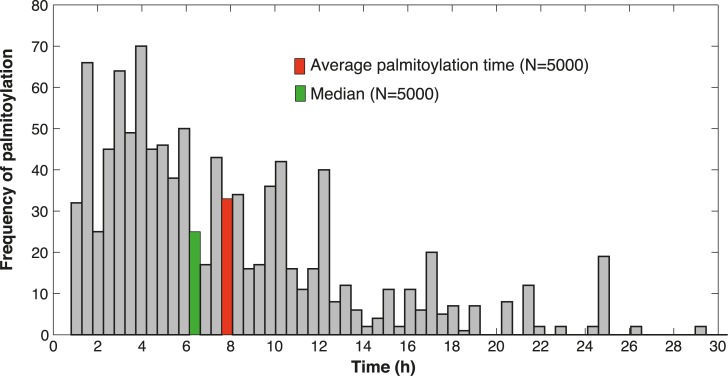
Stochastic simulations reveal the average palmitoylation time for calnexin. Single proteins were tracked in 5000 stochastic simulations of the labelling method described in Expanded View. From the simulations we estimated the average and median time requested for a single molecule of calnexin to undergo double palmitoylation.

An unexpected and intriguing observation in this study is the slow appearance of the palmitoylated population following synthesis (Fig 3A) and the absence of a plateau in the palmitate incorporation experiment (Fig 1H). This suggests that there is a lag time between synthesis and palmitoylation of calnexin. To evaluate this lag time, we derived a stochastic formulation of the original model of the palmitoylation process, and performed stochastic simulations that allowed us to track single proteins in the system from synthesis to dual-palmitoylation (Supporting information, S5 Fig). Simulations were performed for 5000 molecules. Of these about 3000 were degraded before any palmitoylation event occurred. For the remaining population, we determined the frequency distribution of the time required to reach dual-acylation (Fig 6). Some molecules underwent palmitoylation within a few hours of synthesis (Fig 6). Most however remained in the non-palmitoylated form for extended periods of time, leading to an average time of synthesis-to-palmitoylation of 8 hrs (Fig 6).

### Phosphorylation is a negative regulator of calnexin palmitoylation

**Fig 7:**
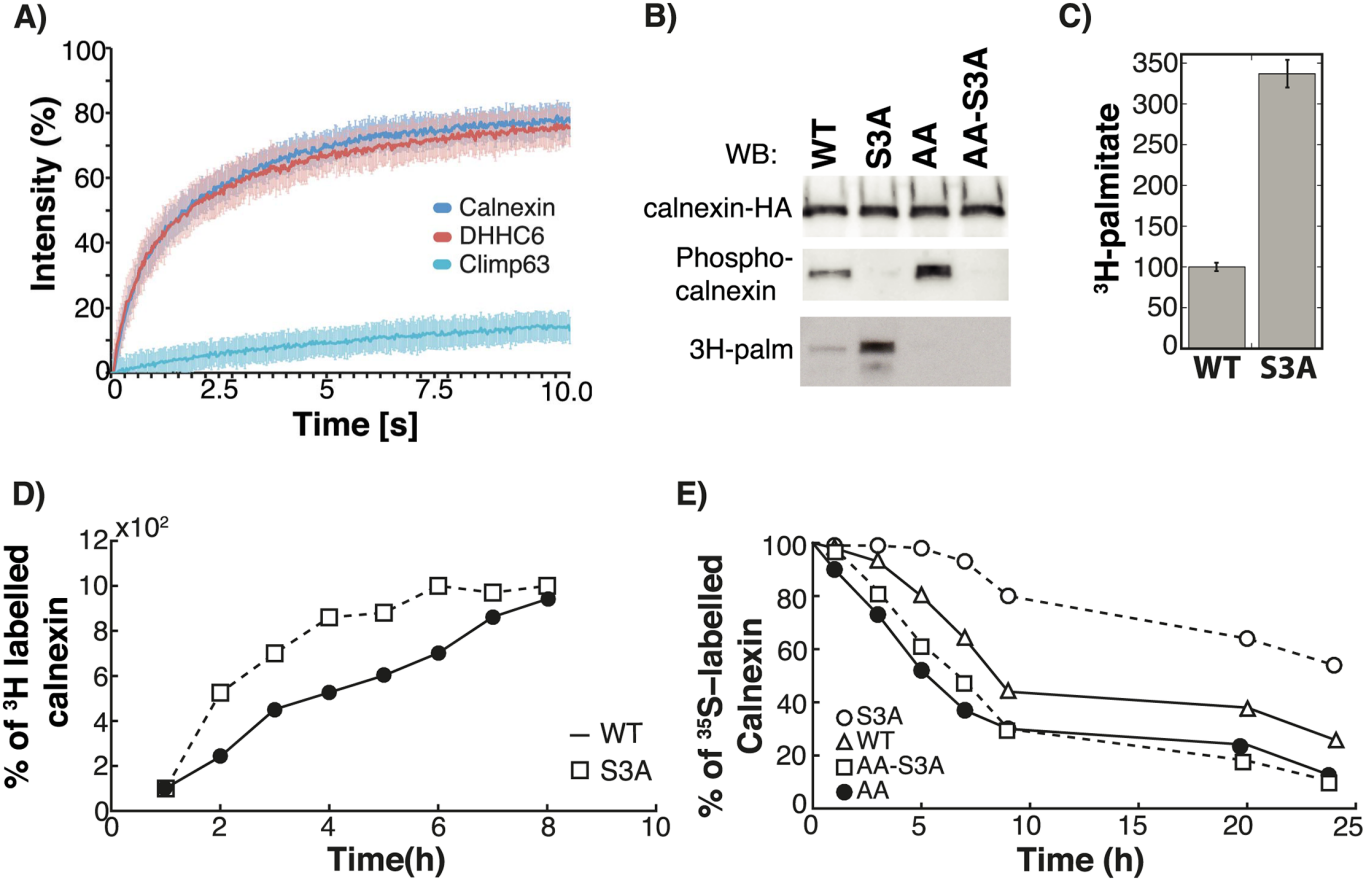
Premature calnexin palmitoylation is prevented by serine phosphorylation. **A:** Hela cells were transfected 48 hrs with Calnexin-GFP, GFP-DHHC6 or GFP-Climp63. Cells were submitted to FRAP analysis (see Material and Methods). **B-E:** HeLa cells were transfected for 24h with calnexin-WT-HA, calnexin-S3A-HA, calnexin-AA-HA or calnexin-AA-S3A-HA. **C:** Cells were incubated with ^3^H-palmitic acid for 2h, washed prior to immunoprecipitation using anti-HA antibodies. Immunoprecipitates were split into two, run on SDS-PAGE and analyzed either by autoradiography (^3^H-palmitate) or Western blotting (anti-HA or anti phospho-calnexin). Autoradiograms were quantified using the Typhoon Imager (Image QuantTool, GE healthcare). Errors correspond to standard deviations (n = 3). **D:** Cells were incubated with ^3^H-palmitic acid for different hours at 37°C, washed prior to immunoprecipitation using anti-HA antibodies. Immunoprecipitates were split into two, run on SDS-PAGE and analyzed either by autoradiography (^3^H-palmitate) or Western blotting and autoradiograms were quantified using the Typhoon Imager (Image QuantTool, GE healthcare). **E:** Cells were incubated 20 min pulse at 37°C with ^35^S-methionine/cysteine, washed and further incubated for different times at 37°C. Calnexin were immunoprecipitated and analyzed by SDS-PAGE. Autoradiography and western blotting were quantified using the Typhoon Imager (Image QuantTool, GE healthcare).

Calnexin and its palmitoylating enzyme DHHC6 are two membrane proteins that reside within the same two-dimensional space of the ER membrane. The lag time between synthesis and palmitoylation of calnexin could potentially be due to slow diffusion of one of the two molecules. We therefore determined their mobility using Fluorescence Recovery after Photobleaching (FRAP) of C-terminally GFP-tagged variants. Both calnexin and DHHC6 showed rapid diffusion with rates of 0.63±0.09 and 0.67±0.13 μm^2^/s respectively, in close agreement with previously published rates for calnexin (Fig 7A) [41]. As a control of a slow diffusing membrane protein [42], we confirmed that Climp63/CKAP4 has a diffusion rate of 0.06±0.01 μm^2^/s (Fig 7A) [42].

Given the high rates of DHHC6 and calnexin diffusion, slow palmitoylation of calnexin cannot be explained by a low probability of encounter between the enzyme and the substrate. This raised the possibility that calnexin palmitoylation is actively prevented and led us to investigate the effect of calnexin phosphorylation on its acylation. We generated a triple mutant in which all three serine phosphorylation sites in the calnexin tail (Ser- 554, 564, 583) were mutated to alanine (S3A mutant). Remarkably, mutation of the serine phosphorylation sites led to a ~200% increase in palmitoylation during the 2h labelling period (Fig 7B and 7C). Also, kinetics of ^3^H-palmitate incorporation, in the absence of protein synthesis, were faster for the S3A mutant than for WT calnexin (Fig 7D). Consistently, ^35^S Cys/Meth pulse-chase experiments revealed that the S3A mutant was far more stable than the WT protein (Fig 7E), in particular due to the disappearance of the initial rapid decay phase, which in WT is due to degradation of the non-palmitoylated species. Altogether these observations indicate that palmitoylation of calnexin is under the negative control of serine phosphorylation.

## Discussion

S-palmitoylation is a post-translational modification that is receiving increasing attention as more and more key cellular events and pathways appear to rely on the reversible acylation of specific proteins [7, 14]. The dynamics of this modification and the regulatory mechanisms are however poorly understood. We have here investigated palmitoylation of the key ER chaperone calnexin [15]. Calnexin shares its ability to promote folding of N-glycosylated proteins with calreticulin, a soluble ER protein. Calnexin in contrast spans the ER membrane and harbors a 90 residue cytosolic tail. While the transmembrane nature of calnexin was initially thought to favor the chaperoning of transmembrane proteins, it is increasingly clear that the transmembrane domain and cytosolic tail confer additional properties and functions to calnexin which involve its palmitoylation [17, 20, 28, 43].

We developed a mathematical model of the palmitoylation cycle that accurately captures the properties of the system as shown by its predictive power. This combination of modeling and experimentation led to a variety of interesting, often unexpected, analyses, predictions and conclusions. First, it highlights the under-appreciated complexity of classical metabolic pulse-chase experiments. What is referred to as WT–of calnexin or any other protein–is in fact a complex population of species, the distribution of which evolves with time. In our model, we have distributed calnexin into 5 species, defined by the folding and palmitoylation status. In future studies we will increase the complexity of the model, including additional species with different types of post-translational modifications, such as phosphorylation on three serines of its cytosolic tail [20], a modification which we find affects its palmitoylation status.

Our analysis also indicates that, while ^35^S Cys/Met labels a well-defined sub-population–the one that has been synthesized by the cell during the pulse–, this is by no means the case when labeling cells with ^3^H-palmitate. Radiolabeled palmitate can indeed be incorporated into any molecule that has an unoccupied palmitoylation site but importantly cannot be incorporated into fully palmitoylated proteins, which in the case of calnexin compose ca. 70% of the total cellular population at steady state. These fully palmitoylated proteins are thus silent in such a ^3^H-palmitate pulse-chase analysis.

Most importantly our model provides unprecedented understanding of the palmitoylation process. In the context of calnexin, we found that the molecule undergoes palmitoylation first on site 1. Once this has occurred, site 2 can be readily modified. There is thus cooperativity between site 1 and 2. Depalmitoylation can rapidly occur if a single site is occupied but removal is drastically slowed down if the two sites are acylated. As a consequence, calnexin is either not modified or acylated on both sites, the single palmitoylated state being barely populated. Importantly, the percentage of calnexin that is modified at steady state can be tuned between 0 to 100% by the activity of the DHHC6 palmitoyltransferase. It has recently been shown that the activity of DHHC6, at least for certain substrates, depends on its association with Selenoprotein K (selK) [44]. Selk was found to be upregulated by ER stress [45], i.e. when increased chaperone activity is necessary, which would lead to an increase in DHHC6 activity and thereby in calnexin protein.

Interestingly, we found that palmitoylation not only affects calnexin function—through association with the ribosome-translocon complex or ER-mitochondrial interaction sites [17, 19, 20, 28]–but has a drastic effect on its stability, increasing the average half-life of the molecules from 5 to 45hrs. Interestingly however, palmitoylation only occurs, on average, 8 hrs after the calnexin molecule has been synthesized. This means that, at the population levels, some 80% of the calnexin molecules that a resting cell synthesizes are degraded before they acquire palmitate and thereby activity. Considering that a resting cell has 500’000 to 1 million copies of calnexin at steady state [37–39], this implies that 2.5 to 5 million copies were actually synthesized. While 80% of these are degraded in resting cells, they can potentially be mobilized if palmitoylation occurred earlier after synthesis.

This unexpected apparent inefficiency for a protein that has no folding problems (Fig 1C), combined with the fact that both calnexin–the substrate–and DHHC6 –the enzyme–rapidly diffuse within the same membrane and thus must have a high probability of encounter, led us to search for a mechanisms that would actively prevent palmitoylation.

Since calnexin is known to undergo serine phosphorylation, we investigated the potential impact of this modification on palmitoylation and found that a serine phosphorylation deficient calnexin mutant undergoes greatly accelerated palmitoylation. Our study thus indicates that cells can post-translationally tune the expression level of calnexin by controlling the kinetics of its palmitoylation via phosphorylation of its cytosolic domain.

In addition to providing important novel insight into the mechanisms by which cells control the level of chaperone activity in the ER, the mathematical model that we have elaborated provides a framework to study palmitoylation of other proteins. In particular, a great variety of type I membrane proteins harbor one or two palmitoylation site in the vicinity of their transmembrane domains. It will be of interest to determine whether the rules of cooperativity and 2 site-requirement revealed by calnexin apply to other proteins and how palmitoylation kinetics of these are controlled. Considering that palmitoylation and depalmitoylation are mediated by enzymes that maybe themselves undergo cycles of palmitoylation and depalmitoylation [46, 47], this post-translational modification is particularly in need of mathematical modeling to understand the complexity of its regulation.

## Materials and Methods

### Cells, antibodies and reagents

Hela cells were grown in complete MEM (Sigma) supplemented with 10% foetal bovine serum (FBS), 2 mM L-glutamine, penicillin and streptomycin.

Rabbit antibodies against calnexin were produced in our laboratory against the C-terminal peptide: CDAEEDGGTVSQEEEDRKPK; anti phospho S583-calnexin were from Abcam (ab58503), anti-HA and anti-HA-agarose conjugated beads were from Roche (Applied Science, IN), protein G-agarose conjugated beads from GE Healthcare, HRP secondary antibodies from Pierce.

### Plasmids and transfection

Human calnexin-HA and human calnexin-C501A-C502A-HA, calnexin-C501A-HA (AC), calnexin-C502A-HA (CA), calnexin S554A-S564A-S583A-HA (S3A), calnexin-C501A-C502A-S554A-S564A-S583A-HA (AAS3A) and human-calnexin-SNAP-HA, dog-HA-calnexin-KDEL and human DHHC6-myc were cloned in pcDNA3 [19]. For control transfections, we used an empty pcDNA3 plasmid. Plasmids were transfected into Hela cells for 24 hrs (2 μg cDNA/9.6 cm^2^ plate) using Fugene (Roche Diagnostics Corporation). To generate GFP fusions, calnexin and DHHC6 were cloned into the peGFP vector and CKAP4 was cloned into the peCE vector.

### RNAi experiments

siRNA against human DHHC6 were purchased from Qiagen (target sequence: gaggtttacgatactggttat). As control siRNA we used the following target sequence of the viral glycoprotein VSV-G: attgaacaaacgaaacaagga. For gene silencing, Hela cells were transfected for 72 h with 100pmol/9.2cm2 dish of siRNA using interferin (Polyplus) transfection reagent.

### Radiolabeling experiments

To follow palmitoylation, calnexin expressing cells were incubated 2h or several hours at 37°C in incubation medium (Glasgow minimal essential medium buffered with 10 mM Hepes, pH 7.4) with 200 μCI /ml ^3^H palmitic acid (American Radiolabeled Chemicals, Inc), washed and incubated different times at 37°C with complete medium prior to immunoprecipitation using anti-calnexin or anti-HA antibodies. Beads were incubated 5 min at 90°C in reducing sample buffer prior to SDS-PAGE. Immunoprecipitates were split into two, run on 4–20% gels and analyzed either by autoradiography (^3^H-palmitate) after fixation (25% isopropanol, 65% H_2_O, 10% acetic acid), gels were incubated 30 min in enhancer Amplify NAMP100 (Amersham) and dried or submitted to Western blotting (anti-calnexin). Autoradiograms and western blotting were quantified using the Typhoon Imager (Image QuantTool, GE healthcare).

For metabolic labeling, Hela cells were transiently transfected (24h) or not with calnexin-HA cDNAs, washed with methionine /cysteine free medium, incubated 20 min pulse at 37°C with 50 μCi/ml ^35^S-methionine/cysteine (Hartman Analytics), washed and further incubated for different times at 37°C in complete medium with a 10-fold excess of non-radioactive methionine and cysteine. Calnexin were immunoprecipitated and analyzed by SDS-PAGE.

Protein synthesis was blocked by 30 min treatment with 10 μg/ml cycloheximide (Sigma) at 37°C.

### Immunoprecipitation

For immunoprecipitations, cells were lysed 30 min at 4°C in IP buffer (0.5%NP40, 500 mM Tris-HCl pH 7.4, 20 mM EDTA, 10 mM NaF, 2 mM benzamidine, and a cocktail of protease inhibitors, Roche), centrifuged 3 min at 2000 g and supernatants were pre-cleared with protein G-agarose conjugated beads and supernatants were incubated 16 h at 4°C with antibodies and beads.

### SNAP labeling

To follow SNAP labeling, calnexin-SNAP expressing cells were incubated 6 hours with 10 μg/ml cycloheximide at 37°C in complete medium, then 30min at 37°C in complete medium with 1 μM SNAP-cell-TMR-star (Biolabs) and cycloheximide, then washed three times and incubated different times at 37°C with complete medium prior lysis of the cells. Total cell lysates were analyzed by SDS-PAGE and the fluorescence was measured using the Typhoon Imager (Image QuantTool, GE healthcare).

### Fluorescence recovery after photobleaching

HeLa cells were seeded on FluoroDish (glass bottom: 0.17mm thickness, from World Precision Instrument Inc. USA) and GFP-tagged proteins (calnexin, DHHC6 and Climp63) were transiently expressed for 24 hours. Fluorescence recovery after photobleaching (FRAP) experiments were performed on a Leica SP5 microscope using a 63x oil-immersion objective (1.4 NA). The microscope was operated using the software supplied with the instrument (LAS AF 2009). The 488nm line of the argon laser was set at 60% output and 100% transmission. During the experiment, the cells were kept in a chamber at 37°C and 5% CO_2_. The pinhole was wide-open. The scanner speed was set at 1400Hz. The digital zoom was set at 6. The detector gain was set at 740V. The frame size was set at 512x32. The resulting scanning time was 32ms per frame. Point bleach measurements were performed and the effective radius of the bleached area was calculated as 0.9μm according to [48]. 50 iterations using 4% transmission of the laser were acquired as pre-bleach reference scans. Then the GFP-tagged proteins localized in the ER were bleached a single iteration for 5ms. Afterwards 500 post-bleach iterations were acquired with same settings as for the pre-bleach acquisitions. FRAP experiments were conducted for each condition on 12 different cells expressing the same level of GFP-tagged proteins. Longer post-bleach acquisitions were conducted for Climp63 to enable a better exponential fitting of the post-bleach curves. Diffusion coefficients were extracted (according to [49]) from the fitting of an exponential equation to the post-bleach recovery curves using a hand-made code in matlab (MATLAB 8.0 and Statistics Toolbox 8.1, The MathWorks, Inc. USA).

## Supporting Information

S1 Fig Results of the GA optimization are plotted on top of the experimental data used as objectives. (G-L): Results of the GA optimization are plotted on top of the experimental data used as validation. **A-F:** In order to estimate the parameters of the model the following set of experiments were used. The algorithm used for estimation is *gamultiobj* from MATLAB’s global optimization toolbox. This algorithm finds the overall minimum of multiple objective functions by minimizing the difference between the output of the model and the experimental data of the calibration set. The goodness of the fitting is visualized plotting the output of the *in silico* experiment obtained with the model on top of the corresponding experimental data: **A:** AC mutant ^35^S decay; **B:** WT ^35^S decay; **C:** WT ^3^H incorporation (data are relative to the t = 1h point); **D:** AC mutant ^3^H decay; **E:** AA mutant ^35^S decay; **F:** WT ^3^H decay. **G-L:** In order to ensure that the model was able to capture behaviors of experiments for which it wasn’t trained we used the following set of experiments to validate the output of the model. These experiments were not used to estimate the parameters of the model, therefore its output is a prediction of the result of the different experiments. This analysis ensures that the model is able to make reliable predictions for different experiments and not only for the ones that were used to estimate the parameters. The goodness of the predictions is visualized plotting the output of the *in silico* experiment obtained with the model on top of the corresponding experimental data: **G:** Calnexin WT PAT6 Overepression ^35^S decay; **H:** Calnexin CA mutant ^35^S decay; **I:** Calnexin CA mutant ^3^H decay; **L:** Calnexin WT PAT6 Silencing ^35^S decay.
(EPS)

S2 Fig Schematic representation of the model and the procedure used for ^35^S pulse-chase experiments simulation. **A:** Model scheme used for ^35^S labelling simulations. The labeled species are marked with *. **B:** Qualitative representation of the method used to perform ^35^S *in silico* labeling experiments. Each simulation is performed in 3 phases. 1) Steady state: the model of the endogenous calnexin is simulated until it reaches steady state. 2) Labeling: the synthesis term is switched towards the labeled protein (marked with *) to simulate the addition of the label for a time that matches the experiments. 3) Analysis: the synthesis term is switched again towards endogenous calnexin to simulate the removal of the labeling. The decay of the labeled species is observed at the same time point of the experiments.
(EPS)

S3 Fig Schematic representation of the model and the procedure used for ^3^H pulse-chase experiments simulation. **A:** Model scheme used for ^3^H labelling simulations. The labeled species are marked with *. **B:** Qualitative representation of the steps used to perform ^3^H in silico labelling experiments. Each simulation is performed in 3 phases. 1) Steady state: the model of the endogenous calnexin is simulated until it reaches steady state. 2) Labeling: the reactions of palmitate attachment are switched towards the labeled protein (marked with *) to simulate the addition of the ^3^H palmitate for a time that matches the experiments. 3) Analysis: the reactions of palmitate attachment switched again towards endogenous calnexin to simulate the removal of the radiolabelled palmitate. The decay of the labeled species is observed at the same time point of the experiments.
(EPS)

S4 Fig Distribution of the labeled species in the different palmitoylation states after 2 hours labeling with ^3^H palmitate. The relative amount of labelled protein with respect to the total pool of calnexin inside the cell (2 hours palmitate labelling) is also shown.
(EPS)

S5 Fig Qualitative representation of the procedure used for protein tracking with stochastic simulations, along with proof of convergence. **A:** Each simulation is performed in 2 phases. 1) Steady state: the model is simulated until the pool of endogenous calnexin is in steady state. 2) Analysis: a single calnexin molecule is added to the pool of labeled protein (marked with *), and its route is recorded at each time step. Analysis of the route allows to calculate the time for going from synthesis to the double palmitoylation state. **B:** The average palmitoylation time measured have converged to 8h after 1100 event of double palmitoylation tracked. We performed 5000 single molecule tracking simulations. During these simulations only 1100 molecule were able to get double palmitoylated before getting degraded. Here we reported the cumulative average palmitoylation time to show that the measured value converged to 8h.
(EPS)

S6 Fig Steady state sensitivity analysis of calnexin WT and mutants. In order to determine to which parameters the steady state of calnexin is most sensitive we performed sensitivity analysis on WT and mutants.
(EPS)

S7 Fig Half life sensitivity analysis of calnexin WT. Experimental data and the analysis of the model parameters have shown how palmitoylation affects stability of calnexin. Sensitivity analysis on *in-silico* ^35^S experiments confirm that the protein half life is strongly influenced by the ability of calnexin of being palmitoylated.
(EPS)

S8 Fig Half life sensitivity analysis of calnexin AA mutant. Sensitivity analysis on *in-silico* ^35^S experiments on the AA calnexin mutant shows that if the protein can’t be palmitoylated, it loses the capabilities to regulate its half-life. The stability of the protein become dependent from the degradation rate of the non palmitoylated state only.
(EPS)

S9 Fig Half life sensitivity analysis of calnexin AC mutant. Sensitivity analysis on *in-silico* ^35^S experiments on the AC calnexin mutant. Results show that a single palmitoylation site is not enough to increase the stability of the protein which remain dependent only from the degradation rate of the non palmitoylated state only.
(EPS)

S10 Fig Half life sensitivity analysis of calnexin CA mutant. Sensitivity analysis on *in-silico* ^35^S experiments on the CA calnexin mutant confirm that the presence of only 1 site isn’t enough to be able to regulate the stability of the protein. If compared with S9 Fig it is possible to see how the two sites are not equivalent, palmitoylation of the first site influence more the stability of calnexin.
(EPS)

S11 Fig Fitting of the data used for calibration and validation after averaging the 383 sets of parameters estimated by the GA. In order to estimate a unique set of parameters for the stochastic simulations the 383 sets of parameter where averaged to obtain a single parameter set that was then converted to stochastic. The figure shows that the averaged set of parameters is still able to reproduce both the calibration and validation data.
(EPS)

S1 Text S1 Text contains detailed information about the model formulation, tQSSA application, parametrization, description of *in-silico* experiments, stochastic model formulation and explanations of the analysis performed on stochastic data. (DOCX)

S1 Table Model reactions. The model of calnexin palmitoylation contains 14 different reactions, describing synthesis, folding, degradation, and the enzymatic reactions of calnexin palmitoylation/depalmitoylation. In the following table we describe in detail how the rates for those reactions are calculated.
(DOCX)

S2 Table Model parameters. The output of GA is a set of optimal solutions, where a solution is a complete set of parameter needed to perform model simulations. From this set we extracted a sub-set of 382 solutions which obtained a GA score better than a set threshold for each objective. During the analysis the model was simulated for each set of parameters of the sub-set. We then reported in this paper the mean of the outputs along with the 1^st^ and 3^rd^ quartile of their distribution.
(DOCX)

S3 Table Mass balance equations. In this model calnexin can exist in 5 different states: unfolded (rCAL), folded (fCAL), palmitoylated only on the first site (c1CAL) or the second (c2CAL), or dually palmitoylated (c12CAL). The following table describes the mass balance for each of these species. The rates of the mass balance of each state are described in detail in S1 Table.
(DOCX)

S4 Table Stoichiometric matrix used for stochastic simulations. In the following table we define the stoichiometry and the directionality of the reactions of the model. Each reaction has a directionality that define which are the reagents and which are the products. In this table each line represents a model reaction, while in the columns we find all the states of the model. For each reaction the states of the model that take part to the reaction as reagents are marked with -1, while the states that participate as products are marked with 1. The matrix that is formed in this way allow to attribute the correct directionality to model reactions during the calculation of the mass balance for each state of the model.
(DOCX)

S5 Table Propensity function used for stochastic simulations. In the first column of the table, each line describes a reaction of the model. To each reaction is associated a rate, in the second column, that describes the probability of that reaction to happen at each time step of the stochastic simulation.
(DOCX)

S6 Table Parameters used for stochastic simulations. The following parameters were obtained through the conversion of the deterministic parameters estimated by the GA (see “Conversion of deterministic parameters to stochastic”).
(DOCX)

S7 Table Experimental data for Figs 1 and 2. Due to the small deviation that characterize the set of experimental data used for generating Figs 1 and 2, the error bars in those figures are not clearly visible. Therefore we report here the numeric data for those experiments.
(DOCX)
