# Supplementary material for: Model-Driven Understanding of Palmitoylation Dynamics: Regulated Acylation of the Endoplasmic Reticulum Chaperone Calnexin
Source: PLoS Comput Biol. 2016 Feb 22;12(2):e1004774. doi: 10.1371/journal.pcbi.1004774 (PMC4765739; doi:10.1371/journal.pcbi.1004774)
Supplement: S1 Text — (DOCX) [file pcbi.1004774.s012.docx]

**Supporting information**

**Core model of calnexin palmitoylation**

The core of our model is based on the well established model of protein phosphorylation described in [[1](#_ENREF_1)]. The set of reactions described in Goldbeter paper was used to describe a single calnexin palmitoylation event. Multiple palmitoylation events are modelled replicating this subunit for each palmitoylation site present on the protein. The complete model of calnexin palmitoylation is shown in Fig. 1A.

Because palmitoylation of the different sites seems to be independent, both the palmitoylation options (palmitoylation of c1 followed by palmitoylation of c2 and vice versa) are included in the model.

In the Goldbeter work, the model was mathematically described using mass action terms. Here, because we are dealing with multiple modification events, and in order to reduce the dimensionality of our system, we choose to describe each enzymatic step using the “quasi-steady state approximation” (QSSA). Although this assumption can be a valid for many enzymatic reactions in vitro, it fails to describe those reactions in which the protein and the enzyme have comparable concentrations, as often happen inside the cell. In fact the validity of QSSA is guaranteed only until, i.e. when the enzyme concentration is low with respect to the substrate $\frac{E_{T}}{K_{M}+S_{T}}\ll1$ [[2](#_ENREF_2), [3](#_ENREF_3)].

In particular we adopted what is called “total quasi-steady state approximation” (tQSSA), well described in [[4](#_ENREF_4)] and [[5](#_ENREF_5)]. This approximation has been successfully used in models describing multiple phosphorylation modification in signaling cascades [[5](#_ENREF_5)]. When we have to deal with cellular processes, Tzafriri et al. [[6](#_ENREF_6)] have shown that the tQSSA approximation is valid also when the enzyme substrate concentrations are comparable [[7](#_ENREF_7)].

**Application of tQSSA to the model of calnexin palmitoylation**

In this section we show the step by step application of tQSSA to the mass action model of calnexin. Initially we demonstrate how tQSSA is applied to a double step palmitoylation, without taking into account depalmitoylation and degradation reactions. This system of palmitoylation is identical to the competitive system described in [[4](#_ENREF_4)], and therefore the exact same procedure can be used to apply tQSSA to the calnexin model.

Specifically, consider the following successive reactions of calnexin modification that lead to the double palmitoylation of calnexin from the folded protein:

|  |  | ((1) |
| --- | --- | --- |

Where fCAL represent folded but not palmitoylated calnexin, c1CAL and c12CAL denote the two different palmitoylation states (single and double palmitoylated calnexin), fCAL(c1)_DHHC6 denotes the complex of non-palmitoylated calnexin with the palmitoyl transferase that can lead into the palmitoylation of the site c1. Similarly c1CAL_DHHC6 denotes the complex of the palmitoyl transferase with the calnexin molecules palmitoylated at the c1 site. This complex can then lead into the palmitoylation of the site c2 and the formation of the dually palmitoylated calenxin, c12CAL. This reaction scheme resembles the system presented in equation 14 of [[4](#_ENREF_4)]. As we can see in both cases we have two substrates (fCAL and c1CAL in our case, S1 and S2 in [[4](#_ENREF_4)]) catalyzed by the same enzyme (DHHC6 or E), therefore the two species are competing to be modified by the enzyme. The only difference in the two reaction scheme is that in our case the product of the first reaction is substrate in the second reaction, but we show that this does not make any difference in the application of tQSSA. The reaction scheme in (1) is governed by the coupled ODEs (2,3,4,5,6) below:

|  |  | ((2) |
| --- | --- | --- |
|  |  | (3) |
|  |  | (4) |
|  |  | (5) |
|  |  | (6) |

Now, in order to apply tQSSA we define new quantities, the total substrates:

|  |  | (7) |
| --- | --- | --- |

We then rewrite equation (2,3,4,5) in terms of total substrates

|  |  | (8) |
| --- | --- | --- |
|  |  | (9) |
|  |  | (10) |
|  |  | (11) |

Equations (8 and 9) and (10 and 11) are identical to equations 21a and 21b of the [[4](#_ENREF_4)]. Then, we apply the quasi-steady-state assumption

|  |  | (12) |
| --- | --- | --- |

which gives

|  |  | (13) |
| --- | --- | --- |
|  |  | (14) |

which is equivalent to 22a and 22b in [[4](#_ENREF_4)].

Substituting (14) into (13) leads to the following equation for complex fCAL(c1)_DHHC6 and then complex c1CAL_DHHC6 can be calculated from (14).

|  |  | (15) |
| --- | --- | --- |

It is easy to notice that equation (15) is identical to equation (23) from [4], therefore solving (15) is equivalent to finding the roots of the third degree polynomial (24) in [[4](#_ENREF_4)]. The polynomial is solved following the exact same procedure as in ([[4](#_ENREF_4)], equations (24,25,26)), finally obtaining:

|  |  | (16) |
| --- | --- | --- |

The system of equations (16) contains in the denominators the terms of competing substrates for the active sites of the enzyme DHHC6. In case of multiple competing species we would have additional terms in the denominator as described by the generalized equation (27) in [[4](#_ENREF_4)].

Equivalently, we derive the reaction rates for the reverse steps of depalmitoylation, catalyzed by a different enzyme, namely acyl protein thioesterases (APT).

Based on the above, we show the equivalent steps of applying the tQSSA in the model of calnexin palmitoylation (Fig.1 main text). The following figure describes the network of calnexin’s palmitoylation/depalmitoylation with elementary reaction steps that include the formation of the intermediary enzyme complexes.

The system of ODEs in (17) below describes fully the model in the figure above:

|  |  | (17) |
| --- | --- | --- |

We now define the total substrates. For each state of palmitoylation, the total calnexin species is the sum of the free substrate and the complexes of the calnexin species to both enzymes (DHHC6 and APT).

|  |  | (18) |
| --- | --- | --- |

Now, we rewrite (17) in function of these new quantities

|  |  | (19) |
| --- | --- | --- |

For the ODE of each complex we sum (as in [[4](#_ENREF_4)]) the corresponding ODEs of the species appearing in (18). Next, we apply the quasi-steady-state assumption

|  |  | (20) |
| --- | --- | --- |

and we derive the following system (21)

|  |  | (21) |
| --- | --- | --- |

It is possible to see that (21) is similar to (22a and 22b) in [[4](#_ENREF_4)] and equations (13 and 14) shown above, but it includes additional terms. In order to derive the tQSSA special care must be taken as stated in [5]. The exact tQSSA for this model does not exist, because the “total substrate” defined in (18) involves some complexes that are not relevant when calculating one of the reaction rates [5]. Therefore, in order to derive tQSSA for this system we need to define the pool for each of these complexes and subtract it from the total substrates [5], as it has been done in (21-24) of [5]. This approximation allows us to match the polynomial derived from (21) with the one in (15), which can then be solved following equations (24,25,26) in [[4](#_ENREF_4)]. The resolution of the polynomial allows to derive the generalized formula for tQSSA (27) in [4], which we used to model our system. The final equations are shown in S1 Table. The same approach shown above have been successfully used to model different network of competitive reactions and double modification mechanism, included the double phosphorylation/dephosporylation of MAPK [[5](#_ENREF_5)], which is very similar to the model presented in this paper.

**Description of the model**

One of the most important improvement in this work with respect to what is observed in the most part of the models describing covalent modification systems, is the introduction of synthesis and degradation terms for protein undergoing post-translational modifications (PTMs). In our opinion these terms help better describe the steady state of a living organism with respect to a closed model [[8](#_ENREF_8)], which will eventually reach a chemical equilibrium.

In this model (Fig. 1A), we assume that calnexin is first synthesized by the ribosome from its mRNA to the corresponding peptide and inserted into the membrane (rCAL). Unfolded calnexin goes through a process of folding, forming folded calnexin, which then shows the two sites available for palmitoylation (fCAL). These two sites can be palmitoylated by DHHC6. The first palmitoylation can occur on both sites; c1CAL and c2CAL denote the two different palmitoylation states. After the first PTM calnexin can undergo another palmitoylation event still catalysed by DHHC6; c12CAL represents the double palmitoylated calnexin. Since palmitoylation is reversible the palmitate can be removed from the two sites. The removal of palmitate requires a different type of enzyme, namely Acyl Protein Thioesterases (APTs).

In order to design our model we took into consideration the following assumption:

1. Calnexin can be degraded in each of its palmitoylation states after folding. During the folding process (rCAL to fCAL) no degradation can occur.
2. Palmitoylation increases the half-life of calnexin (observed through experimental data).
3. DHHC6 is present in low concentrations with respect to its substrate calnexin [[9](#_ENREF_9)].
4. Acyl protein thioesterases (APTs) exist in a comparable amount with respect to calnexin [[9](#_ENREF_9)]
5. The two palmitoylation sites may have a different affinity with respect to the palmitoylation/depalmitoylation enzymes, so we adopted separated Kms for the two sites.
6. The catalytic constant for each palmitoylation site is the same.
7. Both palmitoylation steps are reversible. Depalmitoylation steps are catalysed by an APT.

Despite its simplicity the model has shown to be able to describe the basic mechanisms underlying the observed experimental results (see section “Parameterization of the model”). A detailed account of all reactions and differential equations in the model is given in Tables S1, S2 and S3.

**Parameterization of the model**

Due to the lack of kinetic data on the enzymatic process of palmitoylation, all the parameters of the model were estimated using a genetic algorithm (GA). GA is a heuristic global optimization algorithm that mimics the process of natural selection.

In order to estimate the parameters, multiple datasets coming from experimental results were considered. Time course labeling experiments were performed in order to characterize the dynamics of calnexin synthesis/degradation and incorporation/loss of palmitate. The experimental data used to calibrate the model can be divided in 3 main categories:

1. **^35^S labeling**: cells were treated with ^35^S cysteine/methionine (see material and methods for detailed description) for 20 minutes. Then the labeling was removed and the abundance of labeled calnexin was measured at different time points. These experiments helped to understand the dynamics of protein synthesis and degradation.
2. **^3^H labeling**: cells were treated with ^3^H palmitate (see material and methods for detailed description) for 2 hours. Then the labeling was removed and the abundance of radiolabelled palmitoylated calnexin was measured at different time points. These experiments were made in order to be able to train the model with the dynamics of palmitate incorporation/loss.
3. **SNAP labeling**: This labeling consists of the fusion of 182 residual polypeptides (called SNAP) with the protein of interest. The protein is then expressed in the cell until it reaches physiological levels, and at that point the SNAP can be specifically and covalently labelled with a fluorescent dye for a specific time. The fluorescent dye is then removed from the culture and then is possible to observe the decay of the labeled protein.

To better characterize the enzymatic steps of palmitoylation of each site present on calnexin, those experiments were performed not only on WT but also on mutants lacking one of the two sites, or both. Some of the experiments were also repeated by blocking protein synthesis (using cycloheximide), or in a condition where the removal of palmitate from calnexin was not possible.

In order to estimate the parameters we used the 6 experiments as presented in S1A-F Fig. The rest of the data were used to validate the output of the model and test its prediction capabilities S1G-L Fig. We used the function *gamultiobj* of MATLAB’s global optimization toolbox to perform the parameter estimation. This algorithm finds the overall minimum of multiple objective functions by minimizing the difference between the output of the model and the experimental data of the calibration set (S1A-F Fig.). A complete description of the genetic algorithm can be found in [[10](#_ENREF_10)]. Here we report the main steps of the algorithm:

- The algorithm begins by creating a random initial population set of parameters. Each set of the population is a set of all the modeling parameters. These are used to perform the simulations. In order to generate the initial population the GA require to specify an upper and lower bound for parameters values, GA would then generate an initial population by uniformly sampling random values between the lower and upper bound. Since we knew that calnexin has a concentration in the order of magnitude of we set bounds for the parameter that spanning over estimated calnexin concentration (from ). Once GA has converged to a set of solutions we repeated this process iteratively adjusting each time the bounds on the parameter. In all the iteration each parameter was allowed to vary ±100% with respect to its initial value. This process was executed until we reach a satisfactory match between simulated data and experiments. This procedure was applied to all the parameters except for the degradation rate constant of the different species. In fact this parameter can be estimated from the turnover rate of the different mutants measured experimentally (^35^S pulse chase experiments; Fig. 1 and 2 in the main text):

The value found for degradation rate constant was allow varying ±20% during parameter estimation. We have also considered the experimental errors based on the standard deviation values of the experimental measurements. We should also mention that none of the final estimated parameter values was at the upper or lower bounds used for them during the GA estimation procedure

- At each step, the algorithm uses the sets in the current iteration to create the next population set. To create the new population set, the algorithm performs the following steps:
  - Scores each set of the current population by computing its fitness value.
  - Selects sets, based on their fitness.
  - Produces sets for the next population from the sets of the current population. The new sets are produced either by making random changes to the parameters of a single set (mutation) or by combining the vector entries of a pair of parameter sets (crossover*)*.
- Replaces the current population with the new sets obtained to form the next generation.
- The algorithm stops when one of the stopping criteria is met. These criteria are:
  - The average relative change in the best fitness function value is less than the fitness function tolerance (10^-6^).
  - The number of iterations reaches the value of 10^4^.

The optimization is based on the minimization of a cost function. To assign a score to each individual in the GA population the cost function was computed using each set of parameters generated by the GA to simulate the 6 different experiments present in the calibration set (see “Simulating the labeling experiments” section). The 6 outputs of the model were then compared with the values of the experimental data in the calibration set as follows:

Where is the vector of fitness function values for the set of parameters , is the value of fitness function Saluated for a single experimental result in the calibration set. For each set of parameter the GA computes the fitness value of each experiment in the calibration set as the sum of the squared difference between the experimental measurement and the output of the model for each experimental point in a single experiment. is the number of measurements that were taken in a single labelling experiment, is the number of objectives we want to minimize and is the number of individuals in the GA population.

Because of the presence of multiple objectives there does not exist a single solution that simultaneously optimizes each objective, so the algorithm provides as output a local Pareto set of solutions, which are equally optimal with respect to the fitness function we defined. The solutions in the Pareto set are equally optimal in the sense that for each of them none of the objective functions can be improved in value without deteriorating the quality of the fitness in some of the other objective values. From the Pareto set provided by the GA and in order to be more accurate and to reduce the variability in the output of the model, we selected the ones that fitted best the calibration data. Selection of this subset was done as follow: for each optimized set of parameters, the GA provides the corresponding score for each objective, in our case to each parameter set are associated 6 different scores, one for each calibration dataset used during the optimization. The first step was, for each parameter set in the Pareto, to scale the scores of each calibration dataset with respect to the maximum scores in each objective :

For each set of parameters in the Pareto we then computed the sum of each fitness value to obtain a single score value representing the overall goodness of fit of a set of parameters with respect to the objectives [6]. This value was then scaled to be in the range between 0 and 1 [7]. The sets of parameters with a fitness value of 0.3 or less were selected in the final set of parameters used for simulations and predictions.

The results of the optimization can be found in S2 Table.

.

**Sensitivity analysis:**

Once the calibration of the model was complete we performed parametric sensitivity analysis (SA), with the aim of gaining more insight on how the various parameters of the model affected its output. In particular, we were interested in understanding which parameters are fundamental in determining the abundance and the half life of calnexin. SA was performed using a simple One-at-a-time (OAT) method, which consisted of changing one parameter at a time and observing its effect on the states of the model.

We explain here the step-by-step procedure adopted to calculate sensitivity for each of the 382 sets of parameters. The description refers to the calculation of sensitivity indexes in the case of calnexin’s half-life, but the same procedure applies when calculating sensitivity indexes with respect to protein abundance:

1. We pick a set of parameter from the 382 sets and simulated a ^35^S labelling experiment (See “**Simulating the labeling experiments**” section). In these type of experiments we label calnexin with radioactive amino acids for 20 minutes, we then stop the labelling and measure the abundance of radiolabelled calnexin in the cell at different time points. The half life of calnexin correspond to the time point at which its concentration drop to 50% of the concentration measured at the end of the labelling phase (S7-10 Fig. the graph in the top left corner). The values measured this way will be taken as reference points.
2. Iteratively, each parameter of the set is perturbed (increased) by 1% of its value.
3. After perturbation of a parameter we repeated the simulation of point 1. The values measured this way will be stored as perturbed points.
4. The value of the parameter modified is restored to its original value

Points 2 to 4 are repeated for each of the 17 parameters of a set, resulting in 17 different perturbed simulations. For each simulation only the value of a single parameter is changed.

1. In order to calculate dynamic sensitivity analysis, a sensitivity index for each parameter and each time step of simulation in 1 (and 3) was calculated as follows:

where *S_i_*(*t_j_*) is the sensitivity index of the *i*^th^ parameter at the time step *j*. is the model output at time *t_j_* for the set of parameter *P* in which the parameter *x*_i_ was varied of 1% (point 3). is the model output at time *t_j_* for the set of parameter *P*_r_ used as reference (point 1).  is the *i*^th^ perturbed parameter while is the same parameter but with reference value.

By computing sensitivity for each time step of the simulation, we end up with *j* sensitivity indexes (one for each time step). Since this method was applied for each parameter, we obtained *j ** 17 (the number of parameters in a set) sensitivity indexes. This process was repeated for each of the 382 sets of parameters. The sensitivity indexes calculated for each parameter were than averaged to obtain a single sensitivity index for each time point of the simulation. For each time step standard deviation of sensitivity among the set of 382 parameters was also calculated.

Results of sensitivity analysis on abundance in steady state for WT and each calnexin mutant can be seen in S6 Fig. For each mutant in the top panel we reported the steady state distribution and abundance, in the second panel we found the top 5 sensitivity indexes, while in the last panel we reported sensitivity for all the parameters. S7-10 Fig. show sensitivity towards calnexin’s half life for WT and mutants. We show the measured half-life in the top-left panel, the sensitivity indexes at 50% calnexin concentration are shown in the top right panel. In the bottom left panel we find the top 5 dynamic sensitivities, while in the bottom right panel we find dynamic sensitivities for each parameter.

As can be seen (S6A Fig.) the WT is much more abundant than the mutants (S6B-D Fig.). If we take a look at the top 3 sensitivity indexes for the mutants AA AC and CA, we can see that these show a similar pattern. In fact, the main determinant of calnexin abundance is synthesis rate *vs*. We then find the degradation rate constant of fCAL (*kd1),* followed by the folding rate of calnexin *kf*, or the degradation rate constant of c1 and c2CAL, namely kd2. In these mutants palmitoylation parameters have no effect on protein abundance. This is expected for the AA mutant, which can’t be palmitoylated, but its surprising in the case of the CA and AC mutant, in which one site can still be palmitoylated. Therefore, results suggest that a single modification of calnexin have little to no effect on its abundance. Instead, if we look at the sensitivity results for the WT we can see that among the top 3 sensitivity indexes, in 2^nd^ and 3^rd^ position we find the degradation rate constant of c12CAL (*kd3*) and the maximum speed of palmitoylation (*Vf*). Since analysis of the steady state concentration have shown that the WT is much more abundant than mutants, sensitivity analysis confirm that palmitoylation parameters are strikingly important to reach such levels of concentration. It is important to highlight that in WT the enzymatic parameters have an important role in determining its concentration, but the same parameters have almost no effect on CA and AC concentration. This suggest that a single palmitoylation step is not enough to increase calnexin concentration with respect to the AA mutant, which can’t be palmitoylated. Therefore a double modification of calnexin is required to reach the level of abundance of WT.

A similar situation is observed when we calculate sensitivity indexes with respect to calnexin’s half-life (S7-10 Fig.). Again in WT (S7 Fig.) the maximum speed of palmitoylation is among the most sensitive parameters, highlighting the important role of palmitoylation for calnexin in determining both the abundance and the half life of the protein. Looking at the sensitivities for the CA and AC mutants (S9-10 Fig.), it is easy to see that a single palmitoylation modification is not enough to affect significantly calnexin’s half-life (since *Vmax* and the enzymatic parameters of the model have almost no effect in CA and AC mutants, but they show an effect on WT).

**Simulating the labeling experiments:**

In order to compute the fitness function to evaluate the goodness of a given set of parameters, we need to be able to replicate the labeling experiment *in silico*; this because the value of fitness function is computed calculating the difference between the experimental points and the output of the model.

In this section we explain how it is possible to perform *in silico* the different types of labeling experiments described in the previous chapters. In order to be able to perform *in silico* labelling the following assumption on *in vivo* experiments were made:

1. When the labelling start calnexin is in a steady state condition.
2. In ^35^S labeling experiments during the labelling we add an excess of radiolabeled cysteines, which are much more abundant than the cysteines already present inside the cell. This mean that during the labelling time only the radiolabeled cysteine will be used to synthesize new proteins.
3. In 3H labelling experiments during the labelling we add an amount of radiolabeled palmitate which is much more abundant than the pool present inside the cell. This mean that during the labelling time only the radiolabeled palmitate will be used to modify palmitoylation substrates.
4. The pool of non labeled protein is in competition with the tagged pool to get palmitoylated by DHHC6, and the same is true for the different palmitoylation sites present on calnexin. Therefore competition terms between the different species and the different sites have been implemented.

- **^35^S labeling**: when we perform this kind of labeling we are introducing in the cell an excess of radiolabelled amino acid. This mean that when the label is present in the cell, the newly synthesized protein will be tagged with radioactive amino acid, so we will end up with pools of tagged proteins, which can be tracked and quantified. In order to reproduce the same situation *in silico* we first duplicated the number of species in our model (S2 Fig.). One of the two set of species represents the WT endogenous calnexin. The other serves to simulate the pool of the labeled protein (marked with * in S2 Fig.), and represent the pool of labelled calnexin in the different palmitoylation states that will rise in the cell once the labeling is active. There is one synthesis term that can be directed through the WT or the labeling model and serves to switch from the unlabeled to the labeled synthesis for a certain period of time (equal to the labeling time) and then back again to the unlabeled synthesis. In the in vivo process when we shift from the unlabeled to the labeled synthesis, some unlabeled species will still be present in the system and affect the kinetics of the enzymatic reactions. This is captured in the *in silico* labeling process by additional competitive terms in each reaction rate that represent the competition between labeled and non-labelled proteins for each enzymatic reaction. In order to implement correctly the competition between the different species we modified the equations of our model as follows [[4](#_ENREF_4)]:

if we consider the competitive reaction scheme (* indicate labeled species):

we can formulate the following mass action equations:

along with the conservation law:

.

If we set:

And we introduce the new quantities:

We can re-write equations in [9] using tQSSA and implementing competition among the different substrates:

Where and .Once we re-write our model according to the previous kinetic terms we proceed as follows (S2B Fig.):

1. We simulate the model representing WT calnexin in the cell until it reaches steady state.
2. When the system is in steady state we activate the synthesis of labeled protein for an amount of time that matches with the experimental labeling time. Note that due to the excess of radiolabelled amino acid added to the cell, we consider that in this time window there is no synthesis of non-labelled protein.
3. At the end of the labeling time we redirect the synthesis from the labeled system back to the unlabeled WT system and we monitor the concentration of labeled protein at the same time points as the experiment, with time zero (t=0) being the end of labeling time.

- **^3^H labeling**: in this kind of labeling we add to the cell an excess of radiolabelled palmitate. During the labeling the cell will incorporate palmitate, and as results all the protein species undergoing palmitoylation will be tagged with radiolabelled palmitate. Again we will end up with a pool of tagged protein, which can be tracked and quantified, and an unlabeled pool of these species. The difference between ^35^S labeling is that the protein is tagged after it acquires the palmitate and not during its synthesis. Only palmitoylated protein will be tagged. This kind of labeling is more suitable to observe palmitate incorporation/decay dynamics.

The reaction rates used for this type of labeling are similar with the ones used for ^35^S labeling. In this respect, there will be species of labeled and unlabeled palmitoylated protein, appearing as competitive terms in the reaction rates. With the labeling molecule being now palmitate, the pool of tagged calnexin will contain two less species. These will be the species that are not acquiring palmitate (i.e. rCAL and fCAL, as in S3 Fig.).

We then performed the simulations following the same procedure as for ^35^S labeling, taking care of adjusting the labeling time to fit the experimental labeling time of ^3^H-palmitate labeling (2 hours) (S3 Fig.).

**Stochastic simulations**

In order to measure the average palmitolation time of calnexin, we set up a technique that allows us to perform *in silico* single molecule tracking. To be able to perform single molecule tracking, we adopted a method similar to what was done in simulation of ^35^S labeling (see previous chapter). In order to be able to track a single molecule inside the model we set up a stochastic model of calnexin palmitoylation (see below). The structure of the model remains the same as described in the previous chapter for ^35^S labelling (S2 Fig.). We always have the presence of two set of identical species (S5 Fig.). The Initial conditions for the pool of the labeled protein differs from what is described before, in fact in this pool we put just one molecule of calnexin in the unfolded state, which will be the tracked protein. The synthesis term is directed always towards synthesis of endogenous calnexin (S5 Fig.) to ensure that in the pool of labelled protein we will always have a single molecule of calnexin. The two set of species are linked through the implemented competition terms for enzymes substrates as we did for deterministic simulations. We simulated the model until the protein in the labelled pool was degraded, and for each time step of the simulation we recorded the time and the position of the labelled calnexin in the system. The analysis of its route allowed us to determine average palmitolation time of calnexin. We repeated this procedure for 5000 molecules in order to be able to have a good average (the convergence of the stochastic simulation algorithm can be seen in S5B Fig.).

**Stochastic model formulation:**

During the development of this model we had to deal with one main issue. Calnexin is a very abundant protein in the cell, two big proteome studies [[11-13](#_ENREF_11)] have estimated that calnexin is present in HeLa cell in a number of copies between and . Because we wanted to measure a property of the system in a steady state condition, we had to simulate our model with a number of molecules that matched the values found experimentally. The high number of molecules used, made stochastic simulations computationally expensive. In order to perform simulations in an acceptable amount of time and resources we used the same reduction approach as we did in the deterministic model. We performed stochastic simulations using a model described with tQSSA. Different studies [[14](#_ENREF_14), [15](#_ENREF_15)] have shown that tQSSA is an approximation that can be applied to stochastic models without losing accuracy. Validation results of the method have already been obtained for the Goldbeter switch, MAPK cascade, Michaelis-Menten kinetics and others kinetic models [[16](#_ENREF_16)]. The model that we used is the same as in the deterministic simulations, but as required by the stochastic solver, we provided a representation through a stoichiometric matrix (S4 Table) and propensity function (S5 Table). All computations were performed using MATLAB R2014b. The stochastic simulations were performed using the first reaction method, a variant of the Gillespie algorithm [[17](#_ENREF_17)].

**Conversion of deterministic parameters to stochastic:**

In order to perform stochastic simulations, the parameters of the deterministic model had to be converted in stochastic parameters. This transformation involves a change of units, from concentration to number of molecules. In order to be able to convert the parameters, we did the following assumption:

- The volume of HeLa cells is estimated to be 1540 (from bionumbers database).
- Since calnexin is an ER protein, its concentration depends on the volume of the endoplasmic reticulum (ER). We estimated the ER volume to be approximately 3% of the average cell volume. Therefore the ER volume is equal to: .
- The number of calnexin molecule per HeLa cell is between and [[11-13](#_ENREF_11)]

To verify that our model would give as output a concentration similar to what would be found in HeLa cell we estimated the average calnexin concentration and compared this value with steady state prediction of our model. In order to estimate the concentration of calnexin in the ER we proceeded as follow:

Knowing that the number of molecules () per ER volume () is equal to:

We can convert this value to calnexin concentration () simply dividing it by the Avogadro constant ():

The previous calculation where made considering calnexin to be present in a million copy inside the cell. If we repeat the same calculation for molecules we can easily see that the concentration of calnexin range between in the ER. Our model estimates that in steady state the concentration of calnexin would vary between . Therefore these predictions are in very good agreement agreement with the experimentally estimated values.

In order to perform stochastic simulations, we had to convert the estimated deterministic parameters of the deterministic model into the corresponding models of the stochastic model. Here we report the step-by-step procedure used to obtain the stochastic parameters. The first step was to reduce the 382 sets of parameters (from the 382 models we estimated from the GA procedure) to a single set, and we then perform 5000 stochastic simulations of this model in order to derive the corresponding statistics. While we could derive the equivalent 382 stochastic models, this would further require 5000 simulations for each one of the 382 sets of parameter and it would have been computationally too expensive (we currently perform stochastic simulation of one model (one set of parameters) in 2 days). If the reviewer and the editor require, we could also perform stochastic simulations for some of these 382 sets of parameters.

In order to derive a single set of parameter we averaged the 382 sets of parameter, obtaining in this way a single set of parameters that can be seen in S2 Table. To ensure that this set of parameter was still able to maintain the prediction capabilities shown by the original group of 382 parameter sets we plotted its output against the experimental dataset used for calibration and validation (S8 Fig.). The conversion of the parameter proceeded then as follow:

- Parameter with units :

- Parameter with units

- No conversion is needed for parameter with units

S6 Table show the parameters obtained through the conversion.

Where for each palmitoylation and depalmitoylation step we define the kinetic parameters of the reaction as:

where k_catP6_ is the catalytic constant for DHHC6, while k_catAPT_ is the catalytic constant for APT1. The catalytic constants are assumed to be the same in all the modification steps. K_c1P6b_ and k_c1P6ub_ are the binding and unbinding rate constants of fCAL to DHHC6 respectively. K_c1APTb_ and k_c1APTub_ are the binding and unbinding rate constants of c1CAL to APT1 respectively. In our model we assume that the binding and unbinding rate to DHHC6 and APT can be different for each state of the model.

1. Goldbeter A, Koshland DE, Jr. An amplified sensitivity arising from covalent modification in biological systems. Proceedings of the National Academy of Sciences of the United States of America. 1981;78(11):6840-4. PubMed PMID: 6947258. Pubmed Central PMCID: 349147.

2. Segel LA. On the validity of the steady state assumption of enzyme kinetics. Bulletin of mathematical biology. 1988;50(6):579-93. PubMed PMID: 3219446.

3. Segel LA, Slemrod M. The Quasi-Steady-State Assumption - a Case-Study in Perturbation. Siam Rev. 1989;31(3):446-77. PubMed PMID: WOS:A1989AP45400005. English.

4. Pedersen MG, Bersani AM, Bersani E, Cortese G. The total quasi-steady-state approximation for complex enzyme reactions. Math Comput Simulat. 2008;79(4):1010-9. PubMed PMID: WOS:000262071900019. English.

5. Pedersen MG, Bersani AM, Bersani E. Quasi steady-state approximations in complex intracellular signal transduction networks - a word of caution. J Math Chem. 2008;43(4):1318-44. PubMed PMID: WOS:000255629300003. English.

6. Tzafriri AR. Michaelis-Menten kinetics at high enzyme concentrations. Bulletin of mathematical biology. 2003;65(6):1111-29. PubMed PMID: 14607291.

7. Borghans JAM, DeBoer RJ, Segel LA. Extending the quasi-steady state approximation by changing variables. Bulletin of mathematical biology. 1996;58(1):43-63. PubMed PMID: WOS:A1996TX41000003. English.

8. Palsson BØ. Systems Biology: Simulation of Dynamic Network States: Cambridge University Press; 2011.

9. Nagaraj N, Wisniewski JR, Geiger T, Cox J, Kircher M, Kelso J, et al. Deep proteome and transcriptome mapping of a human cancer cell line2011 2011-01-01 00:00:00.

10. Deb K. Multi-Objective Optimization Using Evolutionary Algorithms: Wiley; 2001.

11. Merrick BA, Dhungana S, Williams JG, Aloor JJ, Peddada S, Tomer KB, et al. Proteomic profiling of S-acylated macrophage proteins identifies a role for palmitoylation in mitochondrial targeting of phospholipid scramblase 3. Molecular & cellular proteomics : MCP. 2011;10(10):M110 006007. PubMed PMID: 21785166. Pubmed Central PMCID: 3205854.

12. Yount JS, Moltedo B, Yang YY, Charron G, Moran TM, Lopez CB, et al. Palmitoylome profiling reveals S-palmitoylation-dependent antiviral activity of IFITM3. Nature chemical biology. 2010;6(8):610-4. PubMed PMID: 20601941. Pubmed Central PMCID: 2928251.

13. Yang W, Di Vizio D, Kirchner M, Steen H, Freeman MR. Proteome scale characterization of human S-acylated proteins in lipid raft-enriched and non-raft membranes. Molecular & cellular proteomics : MCP. 2010;9(1):54-70. PubMed PMID: 19801377. Pubmed Central PMCID: 2808267.

14. Sanft KR, Gillespie DT, Petzold LR. Legitimacy of the stochastic Michaelis-Menten approximation. IET systems biology. 2011;5(1):58. PubMed PMID: 21261403.

15. Rao CV, Arkin AP. Stochastic chemical kinetics and the quasi-steady-state assumption: Application to the Gillespie algorithm. Journal of Chemical Physics. 2003;118(11):4999-5010. PubMed PMID: WOS:000181289000026. English.

16. Macnamara S, Bersani AM, Burrage K, Sidje RB. Stochastic chemical kinetics and the total quasi-steady-state assumption: application to the stochastic simulation algorithm and chemical master equation. The Journal of chemical physics. 2008;129(9):095105. PubMed PMID: 19044893.

17. Gillespie DT. Exact stochastic simulation of coupled chemical reactions. The Journal of Physical Chemistry. 1977;81(25):2340-61.
