# Supplementary material for: Model-Driven Understanding of Palmitoylation Dynamics: Regulated Acylation of the Endoplasmic Reticulum Chaperone Calnexin
Source: PLoS Comput Biol. 2016 Feb 22;12(2):e1004774. doi: 10.1371/journal.pcbi.1004774 (PMC4765739; doi:10.1371/journal.pcbi.1004774)
Supplement: S1 Table — The model of calnexin palmitoylation contains 14 different reactions, describing synthesis, folding, degradation, and the enzymatic reactions of calnexin palmitoylation/depalmitoylation. In the following table we describe in detail how the rates for those reactions are calculated. (DOCX) [file pcbi.1004774.s013.docx]

**Tiziano Dallavilla et al. S1 Table. Model reactions.** The model of calnexin palmitoylation contains 14 different reactions, describing synthesis, folding, degradation, and the enzymatic reactions of calnexin palmitoylation/depalmitoylation. In the following table we describe in detail how the rates for those reactions are calculated.

|  | | |
| --- | --- | --- |
| Reaction | **Forward rate** | **Reverse rate** |
| 1. Synthesis and folding |  |  |
|  | *v1=* | *-* |
|  | *v2=* | *-* |
| 1. Palmitoylation |  |  |
|  | *v3=* | *v7=***$Vb\cdot\frac{\frac{c_{1}CAL}{Kmb_{2}c_{1}}}{1+\frac{c_{1}CAL}{Kmb_{2}c_{1}}+\frac{c_{2}CAL}{Kmb_{2}c_{2}}+\frac{c_{12}CAL}{Kmb_{1}c_{1}}+\frac{c_{12}CAL}{Kmb_{1}c_{2}}+\frac{APT1t}{Kmb_{2}c_{1}}}$** |
|  | *v4=* | *v8=***$Vb\cdot\frac{\frac{c_{2}CAL}{Kmb_{2}c_{2}}}{1+\frac{c_{1}CAL}{Kmb_{2}c_{1}}+\frac{c_{2}CAL}{Kmb_{2}c_{2}}+\frac{c_{12}CAL}{Kmb_{1}c_{1}}+\frac{c_{12}CAL}{Kmb_{1}c_{2}}+\frac{APT1t}{Kmb_{2}c_{2}}}$** |
|  | *v5=* | *v9=***$Vb\cdot\frac{\frac{c_{12}CAL}{Kmb_{1}c_{1}}}{1+\frac{c_{1}CAL}{Kmb_{2}c_{1}}+\frac{c_{2}CAL}{Kmb_{2}c_{2}}+\frac{c_{12}CAL}{Kmb_{1}c_{1}}+\frac{c_{12}CAL}{Kmb_{1}c_{2}}+\frac{APT1t}{Kmb_{1}c_{1}}}$** |
|  | *v6=* | *v10=***** |
| 1. Degradation |  |  |
|  | *v11=* | *-* |
|  | *v12=* | *-* |
|  | *v13=* | *-* |
|  | *v14=* | *-* |
