# Supplementary material for: Model-Driven Understanding of Palmitoylation Dynamics: Regulated Acylation of the Endoplasmic Reticulum Chaperone Calnexin
Source: PLoS Comput Biol. 2016 Feb 22;12(2):e1004774. doi: 10.1371/journal.pcbi.1004774 (PMC4765739; doi:10.1371/journal.pcbi.1004774)
Supplement: S2 Table — The output of GA is a set of optimal solutions, where a solution is a complete set of parameter needed to perform model simulations. From this set we extracted a sub-set of 382 solutions which obtained a GA score better than a set threshold for each objective. During the analysis the model was simulated for each set of parameters of the sub-set. We then reported in this paper the mean of the outputs along with the 1st and 3rd quartile of their distribution. (DOCX) [file pcbi.1004774.s014.docx]

**Tiziano Dallavilla et al. S2 Table. Model parameters.** The output of GA is a set of optimal solutions, where a solution is a complete set of parameter needed to perform model simulations. From this set we extracted a sub-set of 382 solutions which obtained a GA score better than a set threshold for each objective. During the analysis the model was simulated for each set of parameters of the sub-set. We then reported in this paper the mean of the outputs along with the 1^st^ and 3^rd^ quartile of their distribution.

| Parameter | Mean Value | ±s.d. | Range |
| --- | --- | --- | --- |
| [C]^a^/[T]^b^ | 0.63 | ±0.17 | [0.30-0.87] |
| 1/[T] | 2.31 | ±0.34 | [1.14-2.97] |
| 1/[T] | 0.11 | ±0.03 | [0.08-0.16] |
| 1/[T] | 0.07 | ±0.01 | [0.04-0.09] |
| 1/[T] | 0.02 | ±$5\cdot10^{-3}$ | [0.01-0.03] |
| [C]/[T] | 0.76 | ±0.24 | [0.40-1.14] |
| [C]/[T] | 0.10 | ±0.03 | [0.05-0.14] |
| [C] | 7.06 | ±0.83 | [5.74-10.87] |
| [C] | 87.07 | ±17.61 | [51.88-121.46] |
| $Kmb_{2}c_{1}$[C] | 0.55 | ±0.16 | [0.09-0.96] |
| $Kmb_{2}c_{2}$[C] | 0.51 | ±0.20 | [0.03-0.89] |
| [C] | 0.43 | ±0.08 | [0.23-0.54] |
| [C] | 0.31 | ±0.06 | [0.19-0.47] |
| $Kmb_{1}c_{1}$[C] | 43.68 | ±6.80 | [4.98-86.77] |
| $Kmb_{1}c_{2}$[C] | 38.60 | ±21.82 | [1.94-88.13] |
| [C] | 0.01 | ± | [-0.01] |
| [C] | 0.87 | ±0.16 | [0.58-1.32] |

1. The model was calibrated using relative data for protein amounts, therefore the concentration is expressed in arbitrary units
2. The model was calibrated using experiments measured in hours, therefore the time is expressed as hours in the model
