# Supplementary material for: Model-Driven Understanding of Palmitoylation Dynamics: Regulated Acylation of the Endoplasmic Reticulum Chaperone Calnexin
Source: PLoS Comput Biol. 2016 Feb 22;12(2):e1004774. doi: 10.1371/journal.pcbi.1004774 (PMC4765739; doi:10.1371/journal.pcbi.1004774)
Supplement: S3 Table — In this model calnexin can exist in 5 different states: unfolded (rCAL), folded (fCAL), palmitoylated only on the first site (c1CAL) or the second (c2CAL), or dually palmitoylated (c12CAL). The following table describes the mass balance for each of these species. The rates of the mass balance of each state are described in detail in S1 Table. (DOCX) [file pcbi.1004774.s015.docx]

**Tiziano Dallavilla et al. S3 Table. Mass balance equations**. In this model calnexin can exist in 5 different states: unfolded (rCAL), folded (fCAL), palmitoylated only on the first site (c1CAL) or the second (c2CAL), or dually palmitoylated (c12CAL). The following table describes the mass balance for each of these species. The rates of the mass balance of each state are described in detail in S1 Table.

| Mass balance |
| --- |
|  |
|  |
|  |
|  |
|  |
