# Supplementary material for: Model-Driven Understanding of Palmitoylation Dynamics: Regulated Acylation of the Endoplasmic Reticulum Chaperone Calnexin
Source: PLoS Comput Biol. 2016 Feb 22;12(2):e1004774. doi: 10.1371/journal.pcbi.1004774 (PMC4765739; doi:10.1371/journal.pcbi.1004774)
Supplement: S4 Table — In the following table we define the stoichiometry and the directionality of the reactions of the model. Each reaction has a directionality that define which are the reagents and which are the products. In this table each line represents a model reaction, while in the columns we find all the states of the model. For each reaction the states of the model that take part to the reaction as reagents are marked with -1, while the states that participate as products are marked with 1. The matrix that is formed in this way allow to attribute the correct directionality to model reactions during the calculation of the mass balance for each state of the model. (DOCX) [file pcbi.1004774.s016.docx]

**Tiziano Dallavilla et al. S4 Table. Stoichiometric matrix used for stochastic simulations.** In the following table we define the stoichiometry and the directionality of the reactions of the model. Each reaction has a directionality that define which are the reagents and which are the products. In this table each line represents a model reaction, while in the columns we find all the states of the model. For each reaction the states of the model that take part to the reaction as reagents are marked with -1, while the states that participate as products are marked with 1. The matrix that is formed in this way allow to attribute the correct directionality to model reactions during the calculation of the mass balance for each state of the model.
