# Supplementary material for: Model-Driven Understanding of Palmitoylation Dynamics: Regulated Acylation of the Endoplasmic Reticulum Chaperone Calnexin
Source: PLoS Comput Biol. 2016 Feb 22;12(2):e1004774. doi: 10.1371/journal.pcbi.1004774 (PMC4765739; doi:10.1371/journal.pcbi.1004774)
Supplement: S5 Table — In the first column of the table, each line describes a reaction of the model. To each reaction is associated a rate, in the second column, that describes the probability of that reaction to happen at each time step of the stochastic simulation. (DOCX) [file pcbi.1004774.s017.docx]

**Tiziano Dallavilla et al. S5 Table. Propensity function used for stochastic simulations.** In the first column of the table, each line describes a reaction of the model. To each reaction is associated a rate, in the second column, that describes the probability of that reaction to happen at each time step of the stochastic simulation.

| Reaction | Propensity |
| --- | --- |
|  | ** |
|  | ** |
|  | ** |
| $fCAL\to c_{1}CAL$ | **** |
| $fCAL\to c_{2}CAL$ | **** |
| $c_{1}CAL\to fCAL$ | **** |
| $c_{1}CAL\to c_{12}CAL$ | **** |
|  | ** |
| $c_{2}CAL\to fCAL$ | **** |
| $c_{2}CAL\to c_{12}CAL$ | **** |
|  | ** |
|  | **** |
|  | **** |
|  | ** |
| $0\to rCAL*$ | ** |
| $rCAL*\to fCAL*$ | **** |
| $fCAL*\to$ | **** |
| $fCAL*\to c_{1}CAL*$ | **** |
| $fCAL*\to c_{2}CAL*$ | **** |
| $c_{1}CAL*\to fCAL*$ | **** |
| $c_{1}CAL*\to c_{12}CAL*$ | **** |
| $c_{1}CAL*\to$ | **** |
| $c_{2}CAL*\to fCAL*$ | **** |
| $c_{2}CAL*\to c_{12}CAL*$ | **** |
| $c_{2}CAL*\to$ | **** |
|  | **** |
|  | **** |
|  | **** |
