# Supplementary material for: Model-Driven Understanding of Palmitoylation Dynamics: Regulated Acylation of the Endoplasmic Reticulum Chaperone Calnexin
Source: PLoS Comput Biol. 2016 Feb 22;12(2):e1004774. doi: 10.1371/journal.pcbi.1004774 (PMC4765739; doi:10.1371/journal.pcbi.1004774)
Supplement: S6 Table — The following parameters were obtained through the conversion of the deterministic parameters estimated by the GA (see “Conversion of deterministic parameters to stochastic”). (DOCX) [file pcbi.1004774.s018.docx]

**Tiziano Dallavilla et al. S6 Table. Parameters used for stochastic simulations. The following parameters were obtained through the conversion of the deterministic parameters estimated by the GA (see “Conversion of deterministic parameters to stochastic”).**

| Parameter | Value | Units |
| --- | --- | --- |
|  | 18450 | molecules/h |
|  | 2.31 | 1/h |
|  | 0.11 | 1/h |
|  | 0.07 | 1/h |
|  | 0.02 | 1/h |
|  | 22314 | molecules/h |
|  | 2933 | molecules/h |
|  | 206110 | molecules |
|  | 2543152 | molecules |
|  | 16013 | molecules |
|  | 14944 | molecules |
|  | 12559 | molecules |
|  | 8987 | molecules |
|  | 1275827 | molecules |
|  | 1127490 | molecules |
|  | 380 | molecules |
|  | 25372 | molecules |
