# Supplementary material for: Model-Driven Understanding of Palmitoylation Dynamics: Regulated Acylation of the Endoplasmic Reticulum Chaperone Calnexin
Source: PLoS Comput Biol. 2016 Feb 22;12(2):e1004774. doi: 10.1371/journal.pcbi.1004774 (PMC4765739; doi:10.1371/journal.pcbi.1004774)
Supplement: S7 Table — Due to the small deviation that characterize the set of experimental data used for generating Figs 1 and 2, the error bars in those figures are not clearly visible. Therefore we report here the numeric data for those experiments. (DOCX) [file pcbi.1004774.s019.docx]

**Tiziano Dallavilla et al. S7 Table. Experimental data for Fig. 1 and 2.** Due to the small deviation that characterize the set of experimental data used for generating Fig. 1 and 2, the error bars in those figures are not clearly visible. Therefore we report here the numeric data for those experiments.
